# Structures of the inactive and active states of RIP2 kinase inform on the mechanism of activation

**DOI:** 10.1371/journal.pone.0177161

**Published:** 2017-05-18

**Authors:** Erika Pellegrini, Luca Signor, Saurabh Singh, Elisabetta Boeri Erba, Stephen Cusack

**Affiliations:** 1 European Molecular Biology Laboratory, Grenoble, France; 2 University Grenoble Alpes, IBS, Grenoble, France; 3 CNRS, IBS, Grenoble, France; 4 CEA, IBS, Grenoble, France; University College London Institute of Neurology, UNITED KINGDOM

## Abstract

Innate immune receptors NOD1 and NOD2 are activated by bacterial peptidoglycans leading to recruitment of adaptor kinase RIP2, which, upon phosphorylation and ubiquitination, becomes a scaffold for downstream effectors. The kinase domain (RIP2K) is a pharmaceutical target for inflammatory diseases caused by aberrant NOD2-RIP2 signalling. Although structures of active RIP2K in complex with inhibitors have been reported, the mechanism of RIP2K activation remains to be elucidated. Here we analyse RIP2K activation by combining crystal structures of the active and inactive states with mass spectrometric characterization of their phosphorylation profiles. The active state has Helix αC inwardly displaced and the phosphorylated Activation Segment (AS) disordered, whilst in the inactive state Helix αC is outwardly displaced and packed against the helical, non-phosphorylated AS. Biophysical measurements show that the active state is a stable dimer whilst the inactive kinase is in a monomer-dimer equilibrium, consistent with the observed structural differences at the dimer interface. We conclude that RIP2 kinase auto-phosphorylation is intimately coupled to dimerization, similar to the case of BRAF. Our results will help drug design efforts targeting RIP2 as a potential treatment for NOD2-RIP2 related inflammatory diseases.

## Introduction

NOD1 and NOD2 (Nucleotide-binding oligomerization domain-containing proteins 1 and 2) are cytosolic receptors of the innate immune system, which respond to intracellular fragments of the bacterial peptidoglycans, D-glutamyl-meso-diaminopimelic acid (iE-DAP) and muramyl dipeptide (MPD), respectively [1–5]. Upon ligand recognition, NOD receptors oligomerise and recruit an adaptor protein, Receptor Interacting protein kinase 2 (RIP2), through interactions of their respective CARD domains [6]. RIP2 also contains an N-terminal dual-specificity ser/tyr kinase domain (RIP2K), which belongs to the TKL kinase family (Tyrosine kinase-like) [4, 7]. RIP2 undergoes auto-phosphorylation and K-63 linked ubiquitination at Lys209, becoming a scaffolding protein for downstream effectors. The NOD2-RIP2 complex then triggers a pro-inflammatory signalling pathway which includes induction of autophagy and nuclear factor κB (NF-κB) and mitogen-activated protein kinases (MAPKs) dependent gene expression [6, 8, 9]. In this scenario, auto-phosphorylation of the RIP2K Activation Segment (AS) [10] is important in stabilizing the protein itself, for mediating ERK MAPK signalling and to regulate the NF-κB response [11–15]. Moreover, RIP2 tyrosine kinase activity has also been proposed to play a direct role in activating NOD-dependent autophagy [8, 16, 17].

Aberrant NOD2-RIP2 signalling leads to severe inflammatory diseases. Loss-of-function single nucleotide polymorphisms (SNPs) in NOD2 are associated with the gut inflammatory Crohn’s disease [18], whereas gain-of-function SNPs cause non-intestinal auto-inflammatory diseases, such as Blau syndrome and Early Onset Sarcoidosis [19–24]. Dysregulation of NOD2-RIP2 signalling may be also involved in other diseases such as asthma, colorectal cancer and multiple sclerosis, as suggested by animal models and associative studies [6, 25–28]. Therefore, the NOD2-RIP2 signalling pathway has become an attractive pharmacological target for multiple diseases. Notably, RIP2 involvement distinguishes NOD signalling from Toll-like receptor (TLR) signalling and is required to mediate all *in vivo* host responses to MPD [29, 30]. This makes RIP2 a promising drug target for the NOD2-RIP2 dependent inflammatory diseases, with a particular focus on its kinase domain (RIP2K).

Protein and lipid kinases form one of the most important protein classes targeted for treating human disorders. Indeed, protein kinases in both the active or inactive state have been successfully targeted in inflammatory diseases and cancer. Eukaryotic protein kinases have a bi-lobal architecture, with the catalytic machinery and the ATP binding site located in the cleft between the N- and the C-lobe. Depending on the conformational arrangement of conserved functional motifs, kinase structures are classified to be in the active or inactive state [31–35]. Kinases in the active state are characterised by (i) phosphorylation at one or more serine/threonine residue within the Activation segment (AS), a 20–30 amino acid loop between the two lobes, (ii) N- and C-lobe domain closure to form a proper ATP binding cleft (iii) the orientation of Helix αC into the IN position, allowing formation of a salt bridge between an invariant lysine from β-strand 3 and an invariant glutamate from the Helix αC (Lys72 and Glu91 in PKA) (see S1 Fig for an annotated alignment), (iv) the tripeptide DFG in the IN position, with the conserved aspartate (Asp166 in PKA) chelating Mg^2+^ and being a proton acceptor, and (v) correct orientation of two important intramolecular residue networks known as the Regulatory (R-) and Catalytic (C-) spines [31–33, 36]. The conformational changes required to switch from the inactive to active state can be promoted by several events, such as phosphorylation at the AS, allosteric conformational changes induced by homo-dimerization or interaction with other proteins [33, 34]. In the particular case that the kinase undergoes auto-phosphorylation, a third state has recently been proposed: the ‘prone-to-autophosphorylation’ conformation, which represents the intermediate step between inactive and active states [35]. A kinase in this state is able to phosphorylate itself in *cis* or *trans* despite the absence of phosphorylation of the AS [35]. This conformation is stabilized allosterically by dimerization or by interaction with another protein. Interestingly, all the recently published RIP2K structures, with or without inhibitor bound, show that phosphorylated RIP2K crystallises as a side-by-side dimer, highlighting the possibility that dimerization plays a role in kinase activation [4, 13, 35, 37–40]. The symmetrical RIP2K homodimer is antiparallel with the N-Lobe of one monomer interacting with the C-Lobe of the other and the two active sites facing in opposite directions [38, 39]. Interestingly, BRAF kinase also crystallises in a similar side-by-side dimer and dimerization has been proved to be a key step in RAF kinase activation [41–43]. Indeed, structures of RAF kinases in both the active and inactive states were critical to reveal the significance of dimerization in RAF kinase activation and prompted novel drug design strategies. We therefore aimed to provide a comprehensive study of the activation mechanism of the RIP2K domain, by structural characterisation of RIP2K in the active, inactive and in the prone-to-autophosphorylation states.

Here, we present biochemical, mass spectrometric and structural data on RIP2K in phosphorylated and un-phosphorylated states. Structures obtained show RIP2K in both active and inactive conformation and clarify, the essential requirements for RIP2K activation. Structural data combined with native mass spectrometry (Native MS) and analytical ultra-centrifugation (AUC) show that the active and inactive states correlate with changes in the equilibrium between oligomers in solution. Mutations at the dimer interface severely destabilized RIP2K protein *in vitro*.

## Materials and methods

### Constructs

DNA encoding RIP2K_1-300_ was cloned in pFastBacHTB plasmid (Invitrogen) using *NcoI-HindIII* restriction sites. The baculovirus obtained from this construct has a TEV cleavable His-tag at the N-terminus and contains the spontaneous mutation L294F. The cloning resulted in 4 additional residues (GAMA) being added between the TEV cleavage site and the protein sequence. Protein sequences are reported in S1 Table. To increase the yield, a maltose-binding protein (MBP) tag was introduced using the *In-Fusion* cloning technology (Takara Clontech); this construct has the wild-type sequence. Both constructs (RIP2K and RIPK_L294F_) were used for crystallization trials. Kinetic assays and mass spectrometry (MS) analysis were performed on the RIP2K construct after MBP cleavage. All the mutants described in this work (RIP2K_K47R_, RIP2K_D146N_, RIP2K_R74A_, RIP2K_R74D_ and RIP2K_R74H_) were obtained by PCR mutagenesis of the MBP-RIP2K construct. RIP2_1-540_ was cloned by replacing RIP2K with full-length RIP2 in the MBP-RIP2K construct, using the *In-Fusion* cloning technology.

### Protein expression and purification

Recombinant RIP2K, RIP2K mutants and RIP2 were expressed following a similar protocol. Recombinant baculoviruses were generated using the Bac-to-Bac^TM^ baculovirus expression system (Invitrogen), with minor modifications. Bacmid transfection, virus production, virus amplification and protein expression have been performed at the EMBL Eukaryotic Expression Facility, following guidelines provided by the facility. Briefly, proteins have been expressed in Sf21 cells, using Sf-900 SFM medium (Gibco Life technologies), on shaking at 90 RPM on Sartorius Stedim Biotech "CERTOMAT RM" with a 25mm orbit. Sf21 cells were infected at 0.6×10^6^ cells/ml with a virus dose sufficient to block cell growth one day later. Cells were then harvested 4 days post infection. Cells were re-suspended in Lysis buffer (20 mM Tris pH 7.5, 300 mM NaCl, 500 mM NDSB, 5% glycerol, 2 mM βMe) containing protease cocktail inhibitor (Complete, Roche) and lysed by sonication. After centrifugation at 20,000 ×g for 30 min at 4°C, the supernatant solution was purified according to the tag (cobalt-affinity chromatography or amylose-affinity chromatography). Upon overnight TEV cleavage and dialysis with Buffer A (20 mM Tris pH 7.5, 50 mM NaCl, 50 mM NDSB, 5% glycerol and 1 mM TCEP), the protein was further purified by anion exchange chromatography with a 0 to 1 M NaCl gradient, over two column volumes. The resultant samples were immediately used for the experiments described below and concentrated accordingly.

Dimer interface mutants were purified in slightly different way. Cells were re-suspended in Lysis buffer (20 mM Tris pH 7.5, 150 mM NaCl, 5% glycerol, 2 mM βMe, 0.01% NP40) containing protease cocktail inhibitor (Complete, Roche) and homogenized using a douncer. After centrifugation at 20,000 ×g for 30 min at 4°C, the supernatant solution was purified on amylose-affinity chromatography. Due to protein instability, cleavage of the tag was performed for half an hour at room temperature immediately before MS analysis or kinase activity assay.

Cells containing recombinant RIP2 were re-suspended in Lysis buffer (20 mM Tris pH 7.5, 50 mM NaCl, 2 mM βMe, 0.01%NP40) containing protease cocktail inhibitor (Complete, Roche) and homogenized using a douncer. After centrifugation at 18,000 ×g for 30 min at 4°C, the supernatant solution was purified using amylose-affinity chromatography. Upon overnight TEV cleavage the protein was purified first by anion exchange chromatography as described for RIP2K and second, by size exclusion chromatography in a similar buffer (20 mM Tris pH 7.5, 50 mM NaCl, 0.5 mM TCEP).

### *In-vitro* kinase activity assay

Auto-phosphorylation of recombinant RIP2K and RIP2K mutants was analysed using a radioactive assay. 1.2 μg of RIP2K were mixed with 10 mM MgCl_2_ and 10 μM ATP (10:1 ATP-gamma-P32) at 30°C. Reactions were run for 20 minutes and stopped by adding SDS loading buffer at 1, 3, 5, 10, 15, 20 minutes. Auto-phosphorylation of RIP2K_K47R_, RIP2K_D146N_ and RIP2K dimer interface mutants was analysed by end-point kinetics. 1.2 μg of each mutant were mixed with MgCl_2_ and ATP, with or without 1.2 μg of full length RIP2. The reaction was stopped by adding SDS loading buffer after 120 minutes. Samples were run on a 12% SDS-PAGE gel and results were detected using a Typhoon scanner (GE Health).

### De-phosphorylation and phosphorylation assays

Prior to mass spectrometric analysis, the protein buffer was freshly exchanged against 20 mM Tris pH 7.5, 20 mM NaCl and 1 mM TCEP. Protein de-phosphorylation conditions were established by monitoring the reaction in solution at room temperature by mass spectrometry (see the main text). De-phosphorylation was performed by adding 0.3 U of lambda protein phosphatase (New England Biolabs) to 1 μg of RIP2 for at least 1.5 hours, following by re-phosphorylation in 1 mM ATP and 10 mM MgCl_2_.

### Analysis of intact RIP2K by Liquid Chromatography/Electrospray Ionization Mass Spectrometry (LC/ESI-MS)

LC/ESI-MS was performed on a 6210 TOF mass spectrometer coupled to a HPLC system (1100 series, Agilent Technologies). All solvents used were HPLC grade; the HPLC mobile phases were A: H2O 95%, ACN 5%, TFA 0.03%, B: ACN 95%, H2O 5%, TFA 0.03%. Protein samples were desalted on-line on a C8 reverse phase micro-column (Zorbax 300SB-C8, 5μm, 5x0.3mm, Agilent Technologies) for 3 minutes at 100 μl/min with 100% of mobile phase A, then eluted at 50 μl/min with 70% of mobile phase B. MS acquisition was carried out in the positive ion mode in the 300–3000 *m/z* range and the data processed with MassHunter software (v. B.02.00, Agilent Technologies). The mass spectrometer was calibrated with tuning mix (ESI-L, Agilent Technologies). The mass spectrometer settings were the following: gas temperature (azote) 300°C, drying gas (azote) 7 liters/min, nebulizer gas (azote): 10 psig, Vcap: 4 kV, fragmentor: 250 V, skimmer: 60 V, Vpp (octopole RF): 250 V.

### In gel-digestion and phosphopeptide enrichment by Fe(III)-IMAC

These experiments were performed on RIP2K that was first dephosphorylated and re-phosphorylated *in vitro*. After SDS-PAGE separation, RIP2-containing gel bands were excised and rinsed followed by cysteine reduction, carbamido-methylation and digestion with 12.5 ng/ml of trypsin overnight at 37°C. The peptides were extracted by three washing steps, 20 minutes each, with 5% formic acid (FA) in 50% acetonitrile (ACN) at room temperature. The pooled eluates were concentrated to about 10–20 μl in a SpeedVac centrifuge [44]. Peptides obtained by in-gel digestion were dissolved in 0.1 M acetic acid and loaded very slowly (0.5–2 μL/min) onto the Fe(III)-IMAC column. The flow through of this column was collected, acidified with 5% FA and subjected to sequential solid-phase extraction on R1, R2, R3 Poros resin powder columns. The peptides and phosphopeptides were differentially eluted from the IMAC columns using 1 μL of 20 mg/ml α-CHCA solution in ACN and 5% formic acid (70:30, *v/v*) [45], then 1 μL of mix "α-CHCA solution" and "DHB solution" (i.e., 20 mg/ml DHB solution in ACN and 0.1% trifluoroacetic acid (TFA)in 1:1 ratio (v/v), and finally using 1 μL of "DHB solution" (70:30, *v/v*). The matrix/analyte eluate was spotted on an AnchorChip target (Bruker Daltonics).

### MALDI TOF mass spectrometry

Peptide samples after IMAC enrichment were analysed on a MALDI TOF mass spectrometer (Autoflex, Bruker Daltonics) operated in the reflectron positive ion mode previously calibrated with peptide calibration standard I (Bruker Daltonics) in the 500–5000 *m/z* range. The mass spectra were acquired, and the data were processed with Flexcontrol and Flexanalysis software (v. 3.0) (Bruker Daltonics, Bremen, Germany). Peptides were identified by Mascot server search against RIP2K sequence and using the following parameters: trypsin enzyme, missed cleavages: 3, modifications: methionine oxidation, cysteine carbamido-methylation, serine and threonine phosphorylation, singly protonated monoisotopic mass (M+H)^1+^, peptide tolerance: 0.2 Da.

### Native mass spectrometry

RIP2K, RIP2K_D146N_RIP2K_K47R_ were analysed by native MS in the concentration range of 3–5 μM [46]. RIP2K was de-phosphorylated and re-phosphorylated again prior to be analysed by native MS. Protein buffer was freshly exchanged to 250 mM ammonium acetate with 1 mM DTT. Protein ions were generated using a nanoflow electrospray (nano-ESI) source. Nanoflow platinum-coated borosilicate electrospray capillaries were bought from Thermo Electron SAS (Courtaboeuf, France). MS analyses were carried out on a quadrupole time-of-flight mass spectrometer (Q-TOF Ultima, Waters Corporation, Manchester, U.K.). The instrument was modified for the detection of high masses [47, 48]. The following instrumental parameters were used: capillary voltage = 1.2–1.3 kV, cone potential = 40 V, RF lens-1 potential = 40 V, RF lens-2 potential = 1 V, aperture-1 potential = 0 V, collision energy = 30–140 V, and microchannel plate (MCP) = 1900 V. All mass spectra were calibrated externally using a solution of caesium iodide (6 mg/mL in 50% isopropanol) and were processed with the Masslynx 4.0 software (Waters Corporation, Manchester, U.K.) and with Massign software package [49].

### Analytical ultracentrifugation (AUC)

RIP2K was de-phosphorylated and re-phosphorylated again prior to use. RIP2K and RIP2K_K47R_ buffer was freshly exchanged in 20 mM Tris pH 7.5, 50 mM NaCl, 5% Glycerol and 250 μM TCEP. Various protein concentrations from 4 to 18 μM were tested. Sedimentation-velocity experiments were performed for 22 hours at 42,000 r.p.m and 4°C in a Beckman Coulter XL-I, using a rotor Anti-50 and double-sector cells (Nanolytics) of optical path length 12 mm with sapphire windows. Data were obtained by monitoring as a function of the radial position the absorbance at 280, 260 or 300 nm. The analyses were done with the Sedfit software, v14.7g, and figures made with Gussi v1.0.9e, freely available at http://www.analyticalultracentrifugation.com, and http://biophysics.swmed.edu/MBR/software.html, respectively. Partial specific volumes, $\bar{v}$, of 0.732and 0.733 mL g^-1^, for RIP2K and RIP2K_K47R_, respectively, and buffer density, *ρ* of 1.02 g mL^-1^ and viscosity *η*, of 1.96 cp, were calculated from the composition with the programs sedfit and sednterp (http://sednterp-app.sr.unh.edu/). Sedimentation velocity profiles were analyzed in terms of continuous distribution, *c*(*s*), of sedimentation coefficients, *s* [50]. The experimental *s*-values were corrected to *s*_20w_, in the conditions of water at 20°C, and interpreted through the Svedberg equation: *s* = *M* (1 - *ρ* $\bar{v}$) ⁄ (*N*_A_6*π η R*_H_), where *M* is the molecular mass, *N*_A_ the Avogadro’s number, *R*_H_ = *f/f*_min_ *R*_min_, the hydrodynamic radius, with *f/f*_min_ the frictional coefficient and *R*_min_ the radius of the anhydrous volume.

### Crystallization

Crystallization conditions were established by testing several commercial screens at the EMBL High Throughput Crystallization Laboratory (Grenoble, France) using a Cartesian robot. Crystals of RIP2K in complex with adenylyl-methylene-diphosphonate disodium salt (AMPPCP, Sigma-Aldrich) were obtained at 20°C (293 K) by the sitting drop method from solutions containing 5 mg/ml of RIP2K_L294F_ and 2 mM AMPPCP and 5 mM MgCl_2_ equilibrated against Hepes 100 mM pH 7.5, 1 mM MgCl_2_, 2.0 M LiCl and 5% PEG 6000. Crystals of RIP2K mutants were obtained in similar way. RIP2K_D146N_ was mixed with 0.5 mM staurosporine (Sigma-Aldrich) during the dialysis step and the purified sample equilibrated against 0.1 M MES pH6.5, 2M NaCl, 0.1 mM Na(H_2_PO_4_) and 0.1 mM of K(H_2_PO_4_). RIP2K_K47R_ with 2 mM ADP, 10 mM NH_4_F, 2 mM AlCl_3_ and 5 mM MgCl_2_ was equilibrated against 0.1 M Tris pH 8.5, 0.5 mM (NH_4_)_2_SO_4_. Crystals were transferred to a cryo-protection buffer prepared by mother liquor supplemented with 20% (v/v) glycerol, plunged into liquid nitrogen and stored at 70 K.

### Data collection, structure solution and accession numbers

Diffraction data were collected at beamlines ID23-1 [51], ID29 [52] and MASSIF [53] at the ESRF (Grenoble, France). Diffractions limits were 2.0 Å (RIP2K_L294F_-AMPPCP), 2.8 Å (RIP2K_D146N_-staurosporine) Å and 2.6 Å (RIP2K_K47_) and. Data were processed with XDS [54] or iMOSFLM [55], and further analysed using the CCP4 suite [56]. The structures were solved by molecular replacement using PHASER [57]. For the RIP2K_L294F_-AMPPCP structure, BRAF (PDB accession code 2FB8) was used as molecular replacement search model and the RIP2K model so obtained was used to solve all other structures. Refinement was carried out alternately using REFMAC5 [58] and manual rebuilding with COOT [59]. Models were validated using Molprobity [60] from the PHENIX interface and structure figures were produced with PyMol [61]. Data collection and refinement statistics are given in Table 1. Coordinates and structure factors have been deposited in the wwPDB with accession codes: 5NGO, 5NG2 and 5NG3 for RIP2K-AMPPCP, RIP2K_D146N_-STAU, RIP2K_K47R_ respectively.

**Table 1:** Data collection and refinement statistics.

	RIP2K-AMPPCP	RIP2K_D146N_-STAU	RIP2K_K47R_
**Data Collection**			
**Space group**	P4_1_2_1_2	P4_1_2_1_2	P2_1_2_1_2_1_
**Cell dimensions**			
**a,b,c (Å)**	94.21,94.21,201.06	96.08,96.08,202.27	77.00, 103.23, 204.54
**α,β,γ (°)**	90.00, 90.00, 90.00	90.00,90.00,90.00	90.00,90.00,90.00
**Wavelength**	0.976	1.07	0.972
**Resolution (Å)**	47.25–2.00 (2.05–2.00)	48.04–2.8 (2.95–2.8)	92.16–2.6 (2.69–2.6)
**R**_**merge**_	0.035 (0.389)	0.192 (1.144)	0.128 (0.902)
**I/σ**	19.9 (2.9)	8.8 (2.0)	6.5 (1.5)
**Completeness (%)**	92.8 (71.9)	99.9 (100.0)	98.6 (99.5)
**Redundancy**	3.8 (2.5)	7.2 (7.2)	4.0 (4.1)
**Refinement**			
**Resolution (Å)**	2	2.8	2.6
**No. of unique reflections**	54007	22957	47644
**R**_**work**_**/R**_**free**_ **(%)**	17.03/19.14	20.85/22.50	23.09/26.56
**No. of atoms**	4992	4679	9229
**Protein**	4525	4528	9062
**ATP/Mg**^**2+/**^**SB2/STAU/ Glycerol/ SO4/ PO4**	69 (ATP/Mg)	100 (STAU/PO_4_)	110 (SO_4_)
**Water**	397	51	53
***B* factors**	37.98	56.43	70
**Protein**	37.69 (Chain_A)	55.52 (Chain_A)	67.70 (Chain_A)
	37.82 (Chain_B)	57.10 (Chain_B)	67.00 (Chain_B)
			71.54 (Chain_C)
			72.69 (Chain_D)
**ATP/Mg**^**2+/**^**SB2/STAU/Glycerol/ SO**_**4**_**/ PO**_**4**_	43.10–39.79	55.64 (STAU)	106.43 (SO_4_)
	(ATP- Mg^2+^)	112.34 (PO_4_)	
**Water**	44.63	35.07	45.6
**Bond lengths (Å)**	1.337	1.7324	1.435
**Bond angles (°)**	0.041	0.008	0.094
**Validation**			
**Ramachandran outliers**	0.00%	0.00%	0.00%
**Ramachandran allowed**	98.60%	97.60%	96.90%
**Rotamer outliers**	1.20%	1.60%	4.90%
**Clashscore**	1.43	0.87	2.8
**Molprobity score**	0.94	1	1.78

Values in parenthesis refer to high resolution shell.

Validation has been run through the Molprobity interface of Phenix.

## Results

### Production of the RIP2K in phosphorylated and un-phosphorylated forms

To biochemically and structurally characterise RIP2K in both phosphorylated and un-phosphorylated forms, we cloned three constructs comprising RIP2K residues 1–300: wild-type and two predicted kinases-dead mutants, RIP2K_K47R_ and RIP2K_D146N_ [15, 62, 63]. Lys47 and Asp146 correspond to highly conserved residues in most kinases (e.g. Lys72 and Asp166 in PKA, S1 Fig). The former coordinates the α- and β-phosphate groups of the substrate ATP molecule; the latter is part of the HRD motif (HHD in RIP2K), which serves as proton acceptor during catalysis [33].

**Fig 1:**
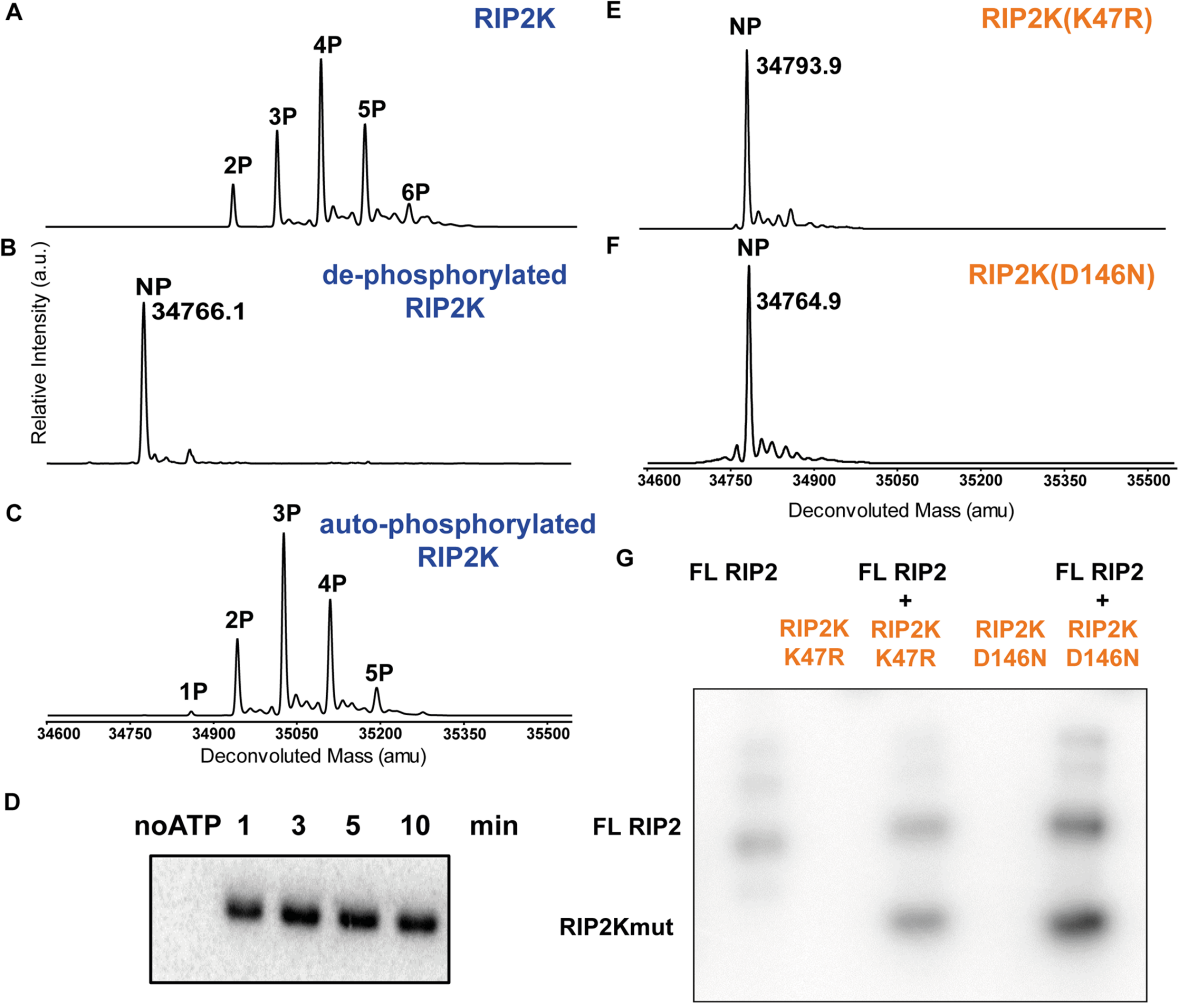
Phosphorylation profile of WT RIP2K and RIP2K mutants using ESI-TOF MS and activity assays. (A-C) MS spectra of RIP2K acquired (A) immediately after its purification from insect cells, (B) after lambda phosphatase treatment and (C) after auto-phosphorylation. Protein sequences used for mass calculations are reported in S1 Table; (D) *In vitro* kinase activity assay of the de-phosphorylated RIP2K using 10 μM ATP. The collected data indicated that RIP2K was completely auto-phosphorylated within 10 minutes. (E-F) MS spectra of (E) RIP2K_K47R_ and (F) RIP2K_D146N_ acquired after their purification from insect cells. The RIP2K mutants were not phosphorylated (NP). (G) *In vitro* end-point kinetic assays of RIP2K mutants at 10 μM ATP, in the presence of recombinant full length (FL) RIP2. RIP2K mutants were *trans*-phosphorylated by FL RIP2, which was also active on itself.

After expression in insect cells and purification to homogeneity, proteins were characterized by mass spectrometry (MS) and by *in vitro* radioactive auto-phosphorylation activity assays (Fig 1). In agreement with previous published data [10, 39] MS analysis indicated that RIP2K was heterogeneously phosphorylated during expression in insect cells (Fig 1A). Prior to *in vitro* kinase assay, RIP2K was treated with lambda protein phosphatase to remove residual phosphorylation and the reaction monitored by ESI-TOF MS. Mass spectra showed that RIP2K was almost completely de-phosphorylated after 90 minutes (Fig 1B). Further de-phosphorylation caused protein precipitation. Auto-phosphorylation was then assayed in the presence of ATP and magnesium (Fig 1C). Compared to the phosphorylation pattern obtained during insect cell expression (Fig 1A), we found a different distribution with a maximum of five phosphorylation sites. The extra phosphorylation site could be related to unspecific phosphorylation activity by insect cell kinases during cell expression. Alternately it could be related to the fact that RIP2K stays in an ATP-rich environment for days instead of minutes, which potentially enhance RIP2K auto-phosphorylation activity. MS data were confirmed by the kinase domain activity (Fig 1D), indicating that de-phosphorylated RIP2K was capable of self-activation.

In contrast, MS data showed that purified RIP2K_K47R_ and RIP2K_D146N_ were not phosphorylated (Fig 1E and 1F). An auto-phosphorylation assay indicated that both proteins were inactive, but they could be phosphorylated *in trans* by purified full length RIP2 (Fig 1G).

### Mapping the phosphorylation sites of RIP2K

RIP2K Activation Segment (AS) comprises residues 167–193 between the conserved DFG and APE (PPE in RIP2K, [64]) motifs (S1 Fig). The AS is rich in possible phosphorylation sites with six serine residues (Ser168, Ser174, Ser176, Ser178, Ser180, Ser181, Ser183), one threonine (Thr189) and one tyrosine (Tyr192) (S2 Fig). In previous studies Ser176 has been identified as a site of auto-phosphorylation [10], while Ser168 has been identified as a possible phosphorylation site through a proteomic approach [65]. To characterise RIP2K auto-phosphorylation sites in the AS and possibly elsewhere, RIP2K was first dephosphorylated and then re-auto-phosphorylated *in vitro* (mass spectrometry profile Fig 1C). After in-gel digestion, phospho-peptides were purified by IMAC [66, 67] and analysed by MALDI-TOF MS (S2 Fig). We identified seven phospho-peptides corresponding to modifications of 172-MMSLSQSR-179 with phosphorylation sites being assigned to Ser174, Ser176 and Ser178. Eight phospho-peptides matched the phosphorylated 172-MMSLSQSRSSK-182 segment and the assignment of the three phosphorylation sites (Ser174, Ser176 and Ser178) was further confirmed. A fourth phosphorylation site detected in 172-MMSLSQSRSSK-182 could correspond to either Ser180 or Ser181. LC-MS/MS analyses on the phosphorylated 172-MMSLSQSR-179 and 172-MMSLSQSRSSK-182 segments further confirmed the assignment of the Ser174, Ser176 and Ser178 modification sites (data not shown). Overall, our data indicate that the RIP2K auto-phosphorylation happens at the AS, on Ser174, Ser176 and Ser178 and on Ser180 or Ser181.

### Structures of WT RIP2K, RIP2K_D146N_ and RIP2K_K47R_

Recently, it has been proposed that auto-activation route of a protein kinase goes through three different conformations: inactive conformation, prone-to-autophosphorylate conformation and active conformation [35]. The prone-to-autophosphorylate conformation is able to bind ATP and to induce the first phosphorylation event at the activation loop. In order to structural define these different conformations in RIP2K, we aimed to crystallize the protein kinase in the three different states.

**Fig 2:**
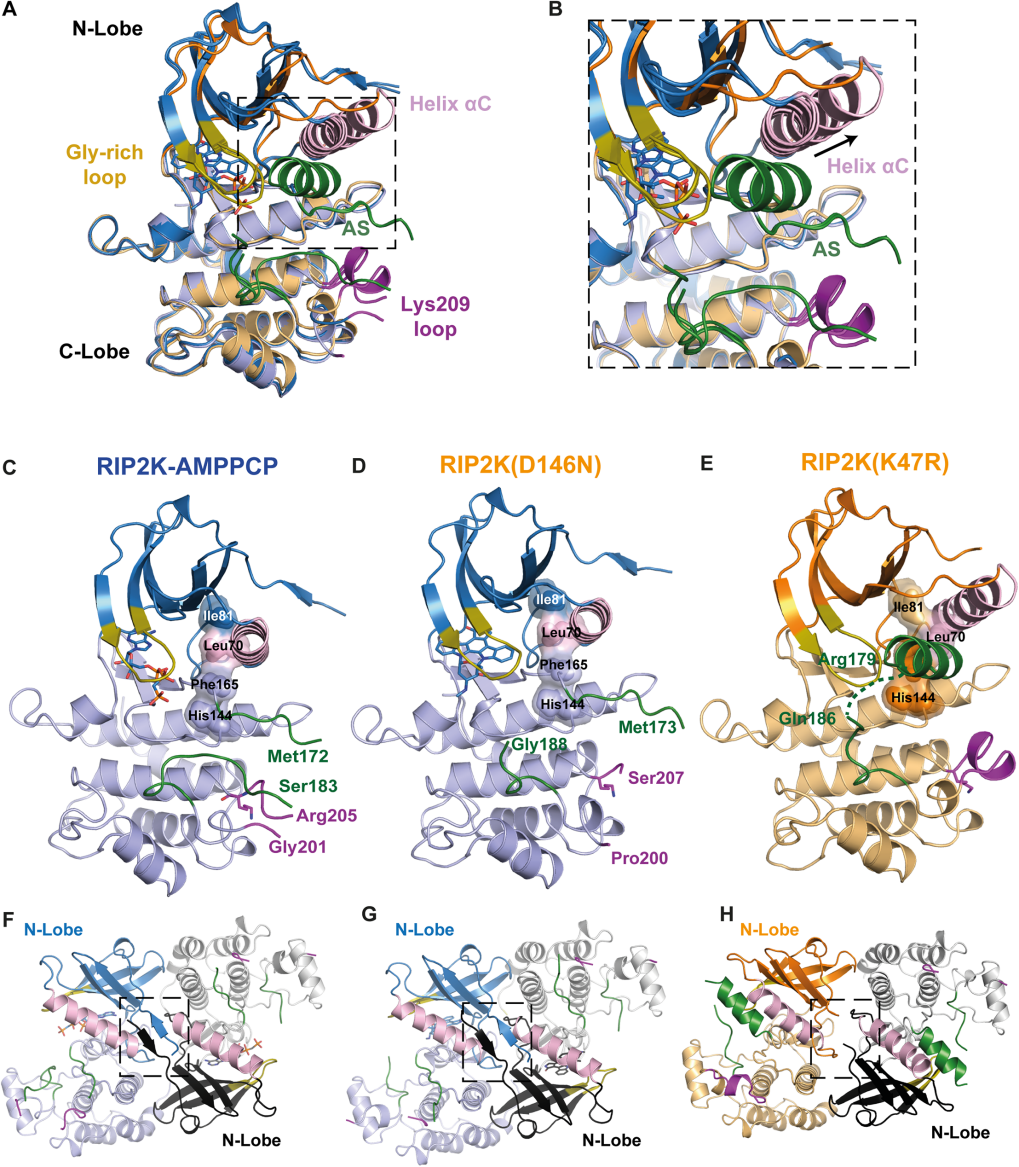
Overview of WT RIP2K, RIP2K_D146N_ and RIP2K_K47R_ structures. (A) Overlay of the ribbon diagrams of RIP2K-AMPPCP, RIP2K_D146N_-STAU and RIP2K_K47R._ (B) The inset highlights the structural differences at αHelix C and at the AS. (C-E) Ribbon diagrams of (C) RIP2K-AMPPCP, (D) RIP2K_D146N_-STAU and (E) RIP2K_K47R_ (E). N- and C-lobes are represented in dark and light colour respectively. The R-spine residues (Ile81, Leu70 from Helix αC, Phe165 from the DGF and His144 from the HHD motif) are shown with surface representation, AS is in green, Lys209 loop in magenta, Gly-rich loop in olive-orange, Helix αC in pink. Lys209 is represented as stick. (F-H) Ribbon diagrams of the side-by-side dimer of (F) RIP2K-AMPPCP, (G) RIP2K_D146N_-STAU and (H) RIP2K_K47R._ The interactions at the N-termini are highlighted. Labelling is kept consistent among the different structures.

As *in vitro* de-phosphorylation causes protein precipitation, we used RIP2K_D146N_ and RIP2K_K47R_ as un-phosphorylated RIP2K samples. We successfully crystallised and solved the structures of all three RIP2K samples (Fig 2): the structure of wild-type RIP2K with bound the ATP analogue AMPPCP (β,γ-methylene-adenosine 5′-triphosphate) (RIP2K-AMPPCP), the structure of RIP2K_D146N_ within Staurosporine bound (RIP2K_D146N_ -STAU) and the structure of RIP2K_K47R_. Data collection and refinement statistics are given in Table 1.

Overlay of the three structures clearly shows that RIP2K-AMPPCP and RIP2K_D146N_ -STAU have similar conformations, while the RIP2K_K47R_ structure shows a different orientation of the Helix αC and consequently of the main functional elements (Fig 2A–2E). All proteins crystallise as side-by-side dimers, as described for previous RIP2K kinases structures. However the new rearrangement of kinase functional elements in the RIP2K_K47R_ structure results in a less extensive dimer interface (Fig 2F–2H). To highlight these differences, we will proceed with the description of each structure obtained, and, by showing how typical kinase functional elements interact with each other at kinase active site and at the dimer interface, we will classify the state of each structure.

### Structures of WT RIP2K and RIP2K_D146N_ are in the active state conformation

**Fig 3:**
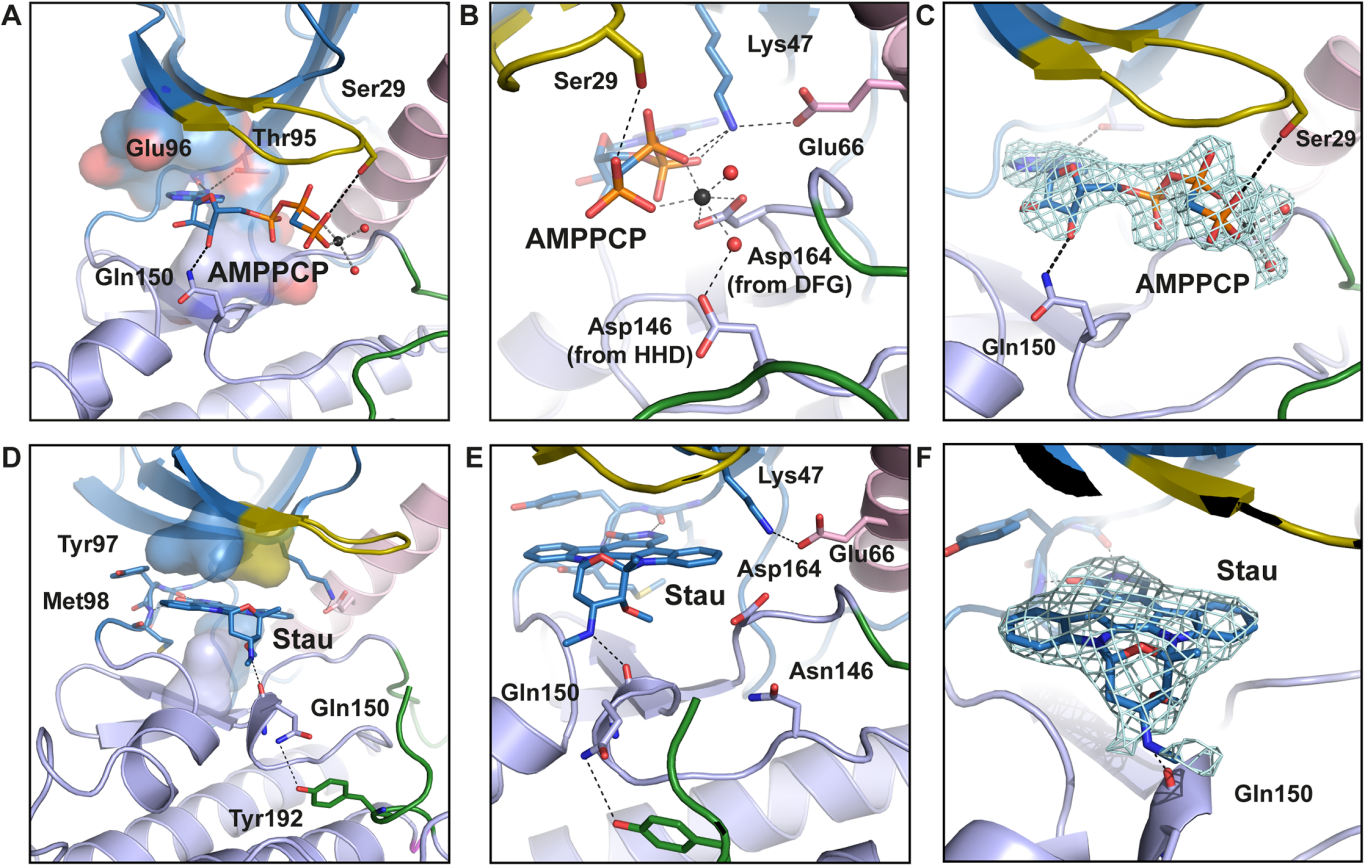
View of the active site of RIP2K-AMPPCP and RIP2K_D146N_-STAU. (A-B) ATP is placed at the interface between the two lobes, within the adenine ring inserted in a pocket formed by residues Ala45, Val46, Leu24, Leu79, Thr95, Tyr97, Leu153 and Ala163 (shown as surface). The adenine ring is hydrogen bonded with the hinge backbone, while N6 atom is coordinated by the hydroxyl group of Thr95 (the gatekeeper residue). The ribose is hydrogen bonded to Gln150, while the ATP-γP is anchored to Ser29, from the Gly-rich loop (residues 25–32). Glu66 in Helix αC forms a salt bridge with Lys47 from strand β3, which then coordinates the ATP-βP and ATP-αP by hydrogen bonds. ATP-βP and ATP-γP are further stabilized by a Mg^2+^ ion, coordinating by Asp164 from the DFG motif and by Asp146 via a water molecule. (C) Fo-Fc map of ATP and magnesium within water molecules is shown as counter level at 1.5 σ. Domain colouring is the same as in Fig 2. (D-F) In the kinase active site, the staurosporine molecule is sandwiched between hydrophobic residues from both N-Lobe (Leu24, Val32 in the Gly-rich loop) and C-Lobe (Leu79, Leu153). The inhibitor molecule is further H-bond to the Hinge backbone and Gln150 backbone. The Glu66-Lys47 salt bridge is shown. (E) Fo-Fc map of staurosporine is shown as contour level of 1 σ. H-bonds are represented in black dash-line. Helix αC is coloured in pink. Metal bonds are in dark grey dash-line. Magnesium is represented as black sphere, water molecules as red spheres.

As expected from the phosphorylation profile, RIP2K-AMPPCP show a typical active state, within AMPPCP bound the active state between the N-and C-lobes (Fig 2C). As we solved the RIP2K-AMPPCP structure at 2.0 Å, significantly higher than the published equivalent one solved at 3.2 Å (PDB: 5AR3, [39]), we will use our structure for comparison purposes. Sequence alignment (S1 Fig) and structural overlay shows the closest similarity with the active states of BRAF (S3 Fig), PKA, CDK2 and EGFR (S2 Table, reviewed in Jura et al., 2011). All essential functional elements are in their canonical positions (Fig 2C). The R-spine is aligned, the DFG motif is in the expected IN conformation, the Helix αC is inwardly displaced [32] and Glu66 in Helix αC (Glu91 in PKA) forms a salt bridge with Lys47 from strand β3 (Lys 72 in PKA) (Fig 3A–3C).

Crystals of RIP2K_D146N_ have been obtained in the apo state and with AMP-PCP or the promiscuous inhibitor staurosporine [68] bound, without significant differences in the resulting structures. The best crystals diffracted to 2.8 Å resolution and were obtained with staurosporine. Each monomer has an inhibitor molecule bound in the active site, whose configuration presents all the features of a kinase in the active state (Fig 2D and 2G). Differently from the ligand in the RIP2K-AMPPCP structure, staurosporine is coordinated by Gln150 backbone, while Gln150 sidechain is hydrogen bonded to the Tyr192 phenyl ring in the AS (Fig 3D–3F). Therefore, the enzyme assumes an active conformation without being phosphorylated, as has been described for IRAK4-KD_D311N_ in complex with staurosporine (PDB code 4U97, [68]). Differently from the IRAK4-KD_D311N_ structure, we did not observe a possible prone-to-autophosphorylate conformation *in cristallo*.

Both the RIP2K-AMPPCP and RIP2K_D146N_ structures exhibit a disordered AS. Only residues 164–172 and 186–196 could be traced for RIP2K-AMPPCP chain B (S4 Fig), implying that there is no electron density for any of the phosphorylation sites detected by mass spectrometry. The AS is close to the loop containing the site of ubiquitination Lys209 (residues 200–210, referred to here as the Lys209 loop), which is also disordered (Fig 2C and 2D). Both structures crystallised in the tetragonal space group *P*4_1_2_1_2 with a dimer in the asymmetric unit, in agreement with the size exclusion chromatography profile (Fig 2D). The dimer is comprised of two monomers arranged side-by-side around a non-crystallographic 2-fold axis and the monomers are quasi-identical (RMSD on Cα is 0.718 for RIP2K-AMPPCP and 0.546 for RIP2K_D146N_ -STAU, calculated with Superpose, [69]). Within the dimer interface, the 2-fold symmetry related N-termini (residues 6–9) form anti-parallel β-strands (Fig 2F and 2G). Despite the fact that Helix J is missing at the C-terminus of our constructs, the dimer is the same as described in other RIP2K structures [38, 39] and also resembles that of BRAF (S3 Fig).

### Structure of RIP2K_K47R_ is in the inactive conformation

Rip2K_K47R_ shows a quite different conformation from RIP2K-AMPPCP and RIP2KD146N-STAU (Fig 2). Previous studies of bone marrow-derived macrophages from RIP2 kinase-dead (K47A) knock-in mice showed defects in signalling, however RIP2 was not expressed at detectable levels suggesting that this inactive kinase was unstable [11]. To avoid this stability problem, we replaced the conserved lysine with an arginine (K47R) instead of an alanine. RIP2K_K47R_ crystallized in a different space group (*P*2_1_2_1_2_1_) with two quasi two-fold dimers (denoted AB and CD) per asymmetric unit and the structure was refined at 2.6 Å resolution. Almost all the functionally important protein segments, except N-terminal residues 1–7 and the tip of the AS (see below) could be traced, in particular for chain A, which is described below. With reference to chain A, chains B,C and D have RSMDs on all C-alpha positions of 0.25, 0.17 and 0.61 Å.

The inactive state of RIP2K resembles the Src/CDK-like inactive conformation, with the DFG motif in the IN state and the Helix αC outwardly displaced (Fig 2A, 2B and 2E) [33, 34]. Helix αC has rotated by ~17 degrees out from its active state position (calculated using the *anglebetweenhelices* command in Pymol) (Fig 2B). Due to the consequent rotation of Leu70, the R-spine is broken. Density for almost all the AS was visible (S4 Fig) showing that it is structured as an α-helix, as described for the inactive states of EGFR-KD and CDK2 (reviewed in Jura et al., 2011[33]) and the monomeric BRAF (S3 Fig) [43]. The same AS Helix, together with the Helix αC inwardly displaced are also present in the structure of the inactive state of RIPK1 bound to an inhibitor (PDB code: 4NEU, [70] and in RIP3K in complex with its substrate MLKL (PDB code: 4M69 [71]. The recent structure of RIP2K in complex with the Type I inhibitor biphenylsulfonamide (PDB code: 5AR8) [39] shows also an outwardly displaced Helix αC, while the AS in the active conformation.

The AS helix encompasses residues 167–178, thus including the phosphorylation sites Ser174, Ser176 and Ser178 while residues 180–184 remain disordered (S4 Fig). The rotation of Helix αC creates space for the helical AS to be packed against it (Fig 2B). The Lys209 loop is also structured as a short α-helix and is more distant from the AS loop than in the active structure. However the same loop is disordered in the other chains of the asymmetric unit. Notably, the 2-fold symmetry related N-termini no longer form anti-parallel β-strands (Fig 2H).

**Fig 4:**
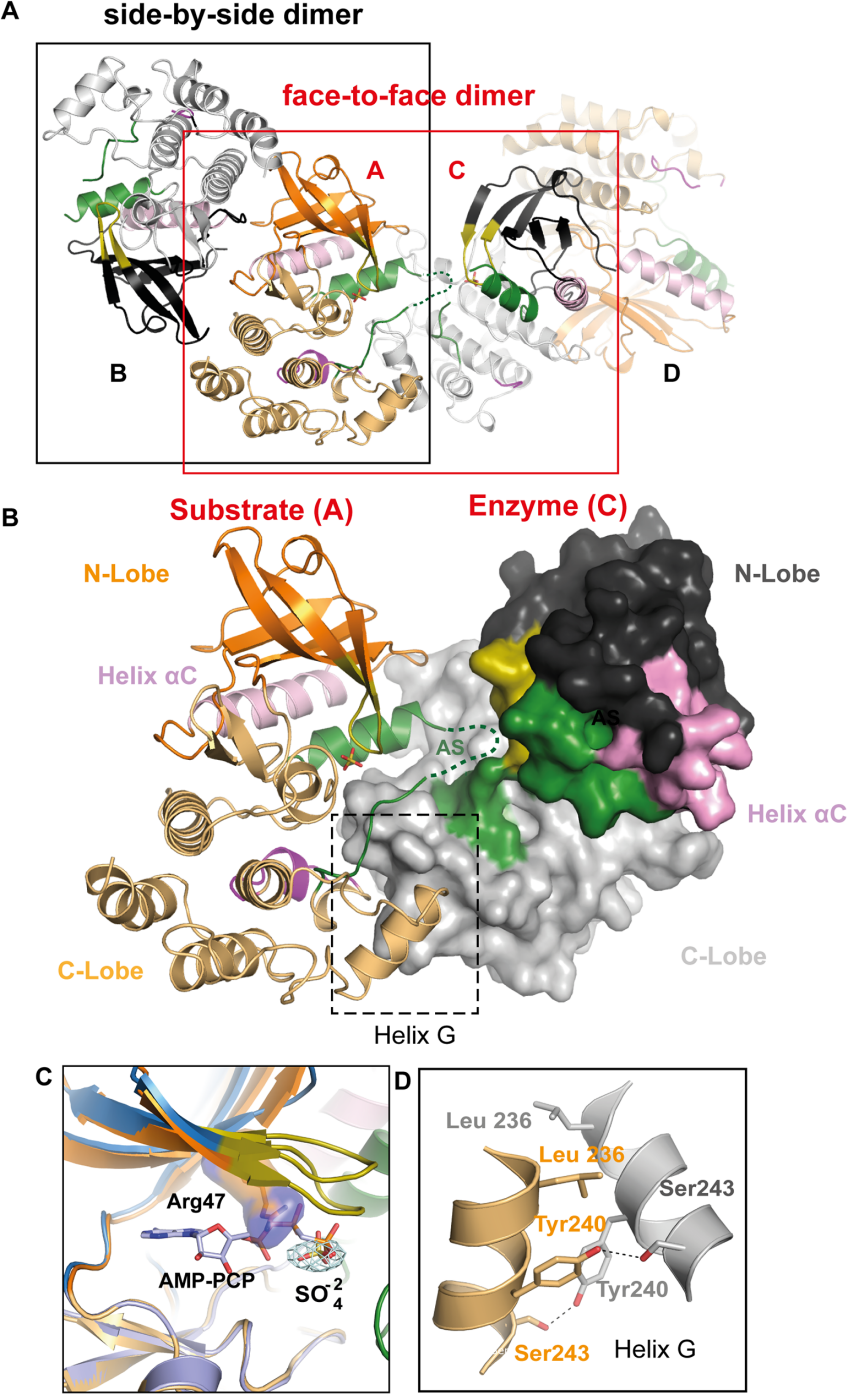
RIP2K_K47R_ asymmetric enzyme-substrate embrace. (A) Ribbon diagram of the Rip2_K47R_ side-by-side dimer and of the face-to-face dimer *in cristallo*. (B) Ribbon diagram of the RIP2K_K47R_ asymmetric, putative enzyme-substrate embrace. The interaction interface is highlighted. (C) Overlay of RIP2K_K47R_ with RIP2K-AMPPCP at the ATP site. The conformation of Arg47 is incompatible with nucleotide binding. Fo-Fc map is shown for the sulphate molecule, at 1.5σ. (D) Interaction at the Helix αG. H-bonds are represented by a black dashed line. Labelling is kept consistent among the different structures.

Interestingly the helical AS from monomer A in the AB dimer projects outwards towards the active site of the asymmetrically related monomer C from the CD dimer, forming a putative substrate-enzyme phosphorylation complex (Fig 4A and 4B). Despite the fact that ADP was added in the crystallisation buffer, no nucleotide is bound, probably due to the bulky arginine blocking the ATP binding site and the sulphate rich crystallisation medium. Indeed both monomers have a sulphate ion bound by Arg47 within the Gly-rich loop and from the overlay with the RIP2K-AMPPCP structure, this ion is at the location of the ATP γ-phosphate (Fig 4C). Interactions between the two molecules involve the C-terminal lobe, with a major involvement of Helix αG which packs asymmetrically on itself (Fig 4D). This asymmetric face-to-face dimer resembles somewhat the asymmetric enzyme-substrate interaction of IRAK4-KD_D311N_ in complex with staurosporine [68], Aurora A in complex with TPX2 [72], and PAK1-KD^K299R/D389N^ [73], which are thought to be close representations of the prone-to-autophosphorylate conformation. However, in contrast to the cited enzyme-substrate interactions, the two helices αE and αF are not involved in the RIP2K asymmetric dimer interaction and neither of the two molecules shows the active conformation.

### Comparison between the active and inactive conformations of RIP2K reveals a critical H-bond network between Helix αC, the AS and the kinase N-terminus

**Fig 5:**
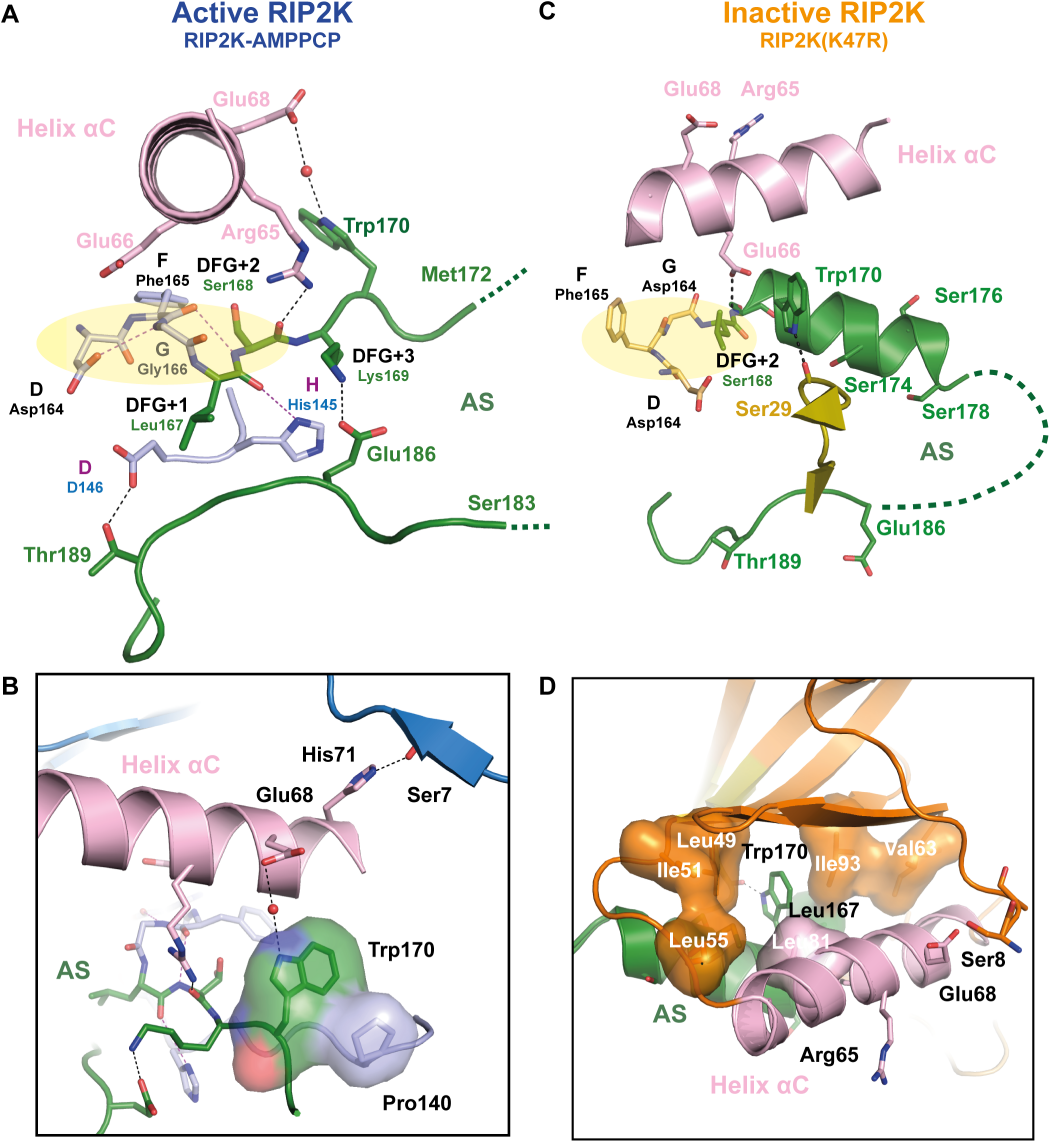
Helix αC, AS and kinase N-termini connections in active and inactive RIP2K. (A-D) Detailed representation of the H-bond network between the Helix αC, AS, magnesium binding loop and N-terminus in (A-B) in RIP2K active state and (C-D) in RIP2K inactive state. For clarity, only one conformation of Ser168 side chain is shown (see S4 Fig). The insets show the interactions of Trp170 and with the N-terminus. Hydrophobic residues are shown as surface. H-bonds are represented as black dashed lines with those related to the DFG-HHD motif are in magenta. The residues belonging to the Magnesium binding loop are highlighted with a pale-yellow ellipse. Hydrophobic interactions are highlighted as surface representation. Missing AS residues are shown with a dashed line.

In the RIP2K-AMPPCP structure, there is an extensive hydrogen-bonding (H-bond) network linking together Helix αC and the ordered part of the AS with their surroundings including the N-terminal anti-parallel β-strands (Fig 5A and 5B; S4 Fig). This network is completely remodelled in the inactive structure (Fig 5C and 5D; S4 Fig).

Proper alignment of the R-spine usually depends on the conformation of the AS together with the magnesium binding loop (which comprises the DFG motif) and the HRD (HHD for RIP2K) motif and importantly the correct orientation of the Helix αC [31, 74] to induce the formation of the Lys-Glu salt bridge. In the RIP2K active conformation (Fig 5A) the side chain of Asp164 (DFG motif) accepts an H-bond from the amide nitrogen of Gly166, whereas the carbonyl oxygen of Phe165 is H-bonded to the amide nitrogen of Ser168 (DFG+2 residue) and His145 (HHD motif) is H-bonded to the carbonyl oxygen of Leu167 (DFG+1 residue). Lys169 (DFG+3) coordinates Glu186, stabilizing the AS conformation. The carbonyl oxygen of the DFG+2 residue (Ser168) is H-bonded to Arg65 from the Helix αC (Fig 5A). This is different from phosphorylated kinases as PKA, where the arginine of the HRD motif (histidine for RIP2K), the DFG+3 residue, and a Helix αC residue are usually hydrogen-bonded to the primary AS phospho-site [74, 75].

Furthermore Glu68 from Helix αC in the active conformation coordinates Trp170 via water molecule (Fig 5A). The tryptophan side-chain makes an edge-to-face interaction with Pro140 and is inserted in a hydrophobic cleft formed by Helix αC (residues 69–73) and residues 138–141 (Fig 5B). Helix αC also interacts with the protein N-terminus: His71 is H-bonded to the Ser8 backbone. Therefore, the active conformation of RIP2K is stabilised by a specific H-bond network which connects Helix αC with the magnesium binding loop, the AS, and the N-terminus without involving any phosphorylation site.

In the RIP2K_K47R_ structure, the reorientation of Helix αC and the AS helix creates an alternative hydrogen bonding network between these two elements, the magnesium binding loop and dimer interface at the N-terminus (Fig 5C and 5D). The salt bridge between Lys47 and Glu66 is broken and, due to the displacement of Helix αC, Glu66 replaces Arg65 in the interaction with the Ser168-Trp170 backbone and N-caps the AS helix. The side chain of Arg65 is flexible and now far from the AS. The amine of the Trp170 indole ring is hydrogen bonded to the backbone carbonyl of Ser29, from the Gly-rich loop (Fig 5C). The Trp170 side-chain is now inserted into a new hydrophobic cleft, formed by Ile93 and Leu167 on one side and Leu81 and Leu49 on the other side. Met173, also from the AS helix, partakes in this new hydrophobic core, interacting with Leu49, Ile51, Leu55 and Val63. It can also be observed that the rotation of Helix αC in the inactive state structure directs Glu68, instead of His71 as in the active state, towards Ser8 in the N-terminal strand (Ser8 only visible in chain A) (compare Fig 5B and 5D). Thus, the asymmetric unit of the RIP2K_K47R_ crystal shows two quasi 2-fold symmetric dimers, with a slightly altered and weaker dimer interface at the N-termini than is observed in the active state.

### RIP2K and RIP2K_K47R_ show differences dimer stability

**Fig 6:**
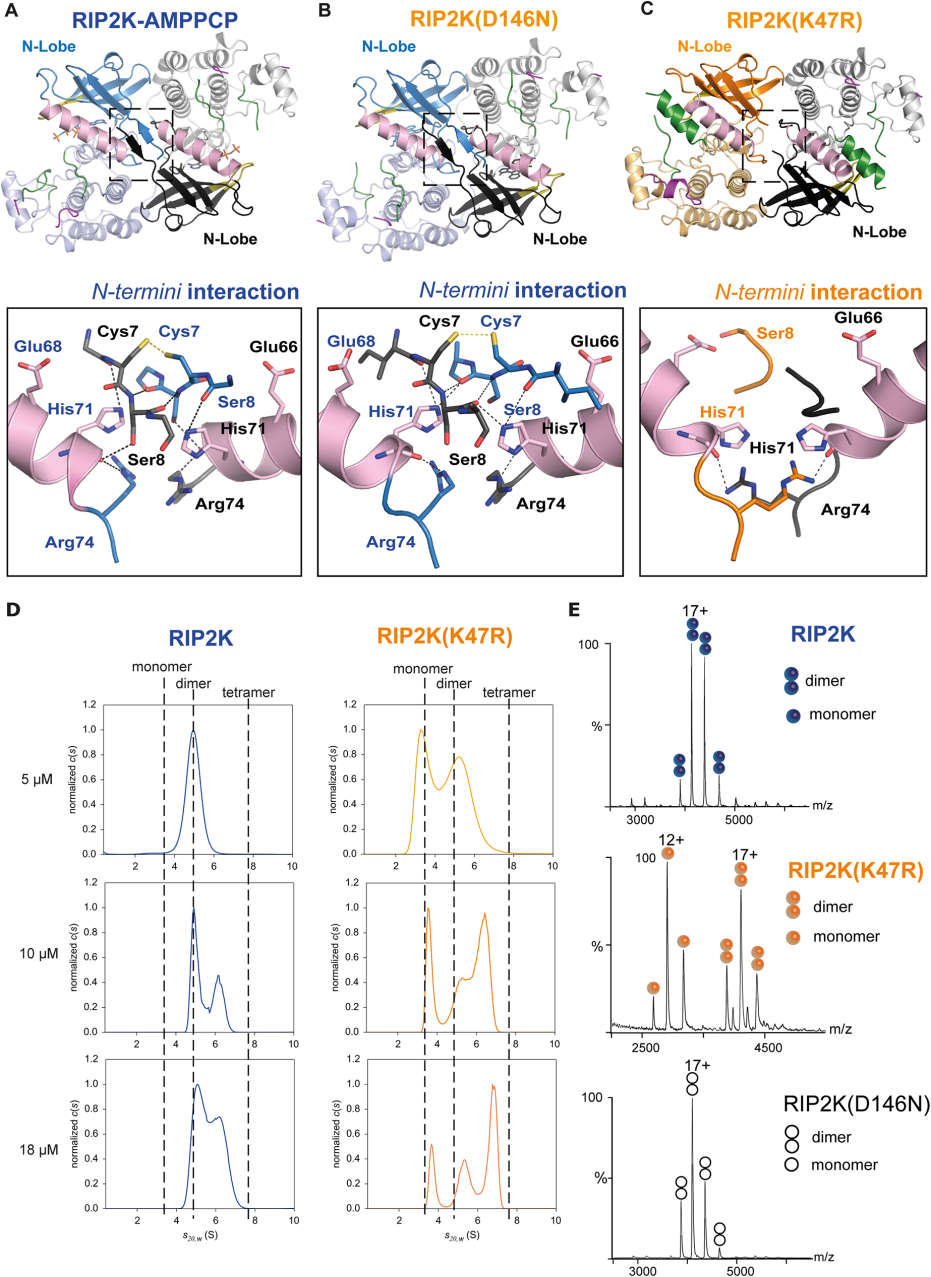
Dimerization of RIP2K and RIP2K_K47R_. (A-C) The insets show the interactions at the N-termini for (A)RIP2K-AMPPCP, (B) RIP2K_D146N_-STAU, (C) RIP2K_K47R_); (C) Sedimentation-velocity AUC analysis of RIP2K and RIP2K_K47R_ at three different protein concentrations. Normalized sedimentation-coefficient distributions are plotted. Non-interacting species analysis of the homogeneous RIP2K at 5 μM showed a unique symmetrical boundary in *c*(*s*), providing independent estimates of *s*_20w_ = 4.9 S and *M* = 66 ± 6 kDa. The latter is close to the theoretical value of 69.6 kDa for the dimer and corresponds to *f/f*_min_ *=* 1.2, indicative of a globular compact shape. Using the same shape factor, we derived *s*_20w_ values of 3.1 S for the monomer, close to the slow boundary observed in RIP2K_K47R_ samples_,_ and 7.8 S for the tetramer, smaller than the fast boundary observed in RIP2K_K47R_ samples, and in RIP2K at 10 and 18 μM, which suggests that the latter is a reaction boundary and corresponds to a mixture of oligomers in equilibrium. (D) Native MS spectra of RIP2K, RIP2K_K47R_ and RIP2K_D146N_-STAU at 5μM. 17+ and 12+ are the main charge states of the dimers and monomers, respectively. The signal intensity represented on the Y-axis is expressed in "arbitrary units".

We find that both RIP2K and RIP2K_K47R_ crystallized as 2-fold symmetric dimers but with slightly different interfaces (Fig 2). In the active conformation, the 2-fold symmetry related N-termini (residues 6–9) form anti-parallel β-strands that are further stabilized by His71 from Helix αC and Arg74 (Fig 6A). Symmetry-related Cys7 residues are positioned close enough to potentially form an inter-molecular disulphide bridge. The RIP2K inactive state dimer has weaker interactions, which correlate with the rearrangement of Helix αC (Fig 6C). Fewer residues at the N-terminal extremity can be traced and chain A shows that outwardly displaced Helix αC interacts with Ser8 using Glu68 as described above. Arg74 interacts with His71 backbone of the 2-fold related molecule, instead of with the one in the same chain, as in the active form.

Taking into account the crystallographic data we further characterised RIP2K dimerization using a combination of AUC and native MS to assess the quaternary structure of RIP2K and RIP2K_K47R_ in solution (Fig 6D and 6E). As the RIP2K dimer interface contains two symmetrically related Cys7 in close proximity, experiments were performed in the presence of DTT or TCEP to avoid disulphide formation. Both techniques confirmed the dimeric state of active RIP2K at low concentration (3–5 μM), whereas the inactive RIP2K_K47R_ mutant is a mixture of monomers and dimers. In both cases increasing protein concentration, promotes oligomerisation, with a special propensity of RIP2K_K47R_ to form tetramers. Tetramers of RIP2K_K47R_ can be seen in *in cristallo*, as face-to-face dimers, where the inner side is represented by the asymmetric RIP2K_K47R_ dimer (Fig 4A). For completeness, we performed native mass spectrometry on the RIP2K_D146N_-STAU sample at 3–5 μM. In agreement with the crystal structure (Fig 6B), the data show that RIP2K_D146N_ is a dimer (Fig 6B and 6E).

**Fig 7:**
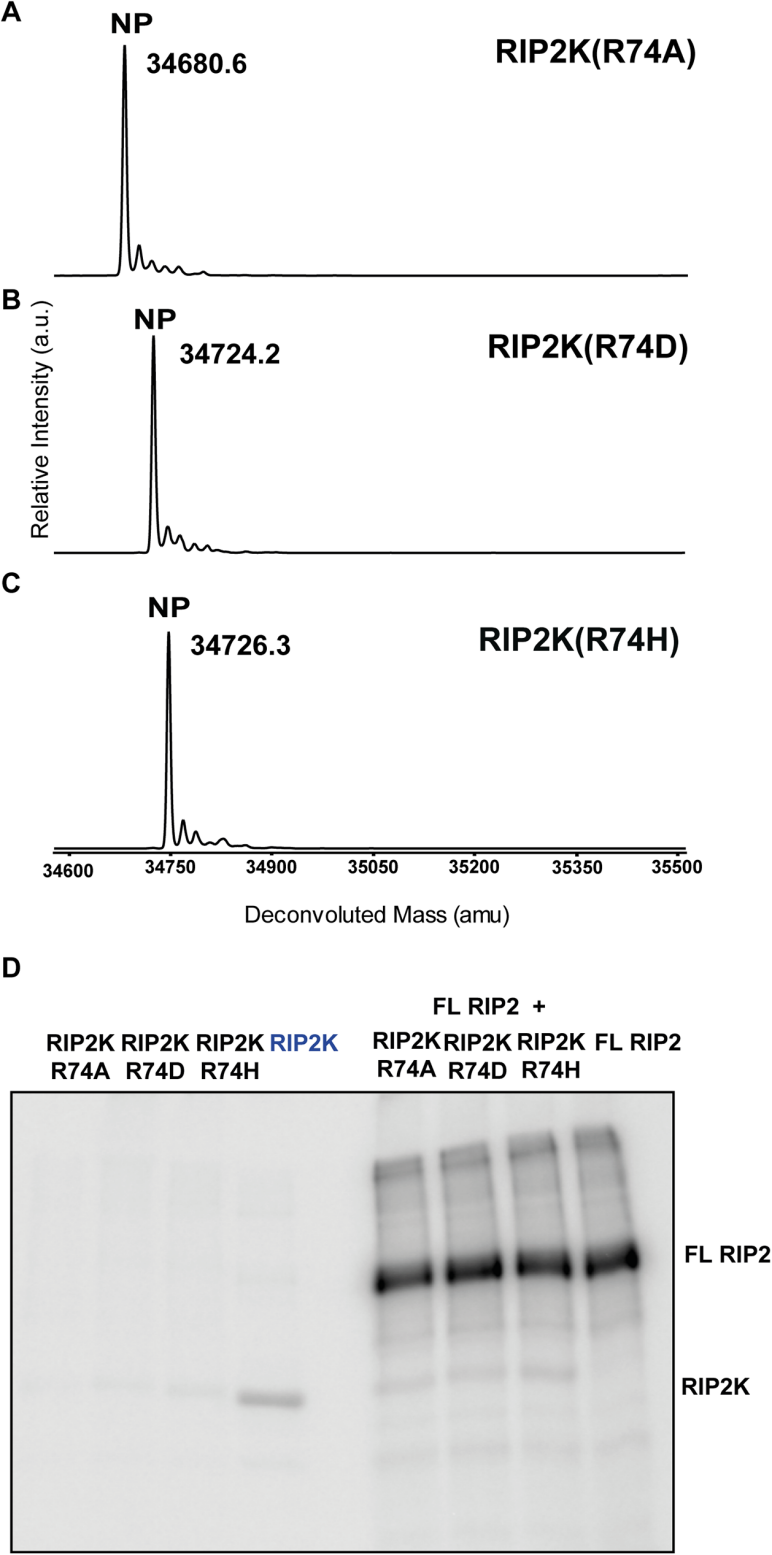
Phosphorylation profile of RIP2K dimer interface mutants using ESI-TOF MS and activity assay. (A-C) MS spectra of RIP2K_R74A_(A),RIP2K_R74D_ (B) and RIP2K_R74H_ (C) acquired immediately after tag cleavage. The dimer interface RIP2K mutants were not phosphorylated (NP). Protein sequences used for mass calculations are reported in S1 Table; (D) *In vitro* end-point kinetic assays of dimer interface RIP2K mutants (RIP2Kmut) and RIP2K at 10 μM ATP, in presence or absence of a recombinant form of full length (FL) RIP2. RIP2K has not previously de-phosphorylated (faint band).

As mentioned in the introduction, active RAF kinases also crystallise as side-by-side dimers. Interestingly the active BRAF kinase dimer shows similar interactions at the N terminus to the RIP2K active dimer (S3 Fig), with BRAF Arg509 playing a similar role to Arg74 in RIP2K. Since Arg509 mutations in BRAF significantly diminish kinase activity by reducing dimer formation [42, 43], we mutated the equivalent Arg74 in RIP2K into alanine, aspartate and histidine. MS analysis showed that RIP2K_R74A_, RIP2K_R74D_ and RIP2K_R74H_ were not phosphorylated (Fig 7A). An auto-phosphorylation assay indicated that all proteins are almost inactive, and in contrast to RIP2K_K47R_ and RIP2K_D146N_, they could not be efficiently phosphorylated *in trans* by purified full length RIP2 (Fig 7B). Analysis by gel filtration chromatography of all three mutants shows that upon concentration the protein aggregates, impeding further analysis by AUC or native mass spectrometry and suggesting an important impact of these mutation on protein stability *in vitro*.

## Discussion

Detailed characterisation of atomic structures has contributed greatly to the success in designing drugs targeting kinases. RIP2, a critical component of the NOD1 and NOD2 signalling pathway, is a promising new target for anti-inflammatory drugs. In the present study we provide a comprehensive structural description of the kinase domain of RIP2 in its active and inactive states. Together with MS, AUC data and mutagenesis experiments, our results provide an overview of the conformational changes necessary to activate RIP2K. The active state of RIP2K is phosphorylated at the AS and is a compact dimer both in solution and in crystals, as seen in this work and other recent publications [38, 39], where an extra helix (Helix αJ) at the C-terminus of the kinase domain has been observed to reinforce the dimerization interface [38, 39]. The inactive state of RIP2K reassembles the Src/CDK-like conformation [33] and forms a less stable dimer in solution. RIP2K kinase dimerization promotes the displacement of Helix αC, with the consequent formation of the Lys47-Glu66 salt bridge and the correct alignment of the R-spine. Kinase activation shifts the oligomerization equilibrium in favour of the compact dimer, which is characterised by a robust β-sheet interaction between 2-fold related N-termini. The importance of the N-termini interaction in kinase activation is highlighted by the dimer interface mutants RIP2K_R74A_, RIP2K_R74D_ and RIP2K_R74H_, which show a dramatic decrease of kinase activation and protein stability *in vitro*. These results are consistent with the hypothesis that Rip2K activation is coupled to dimerization and they suggest that RIP2K dimerization is stabilised by the movement of Helix αC into the active position.

**Fig 8:**
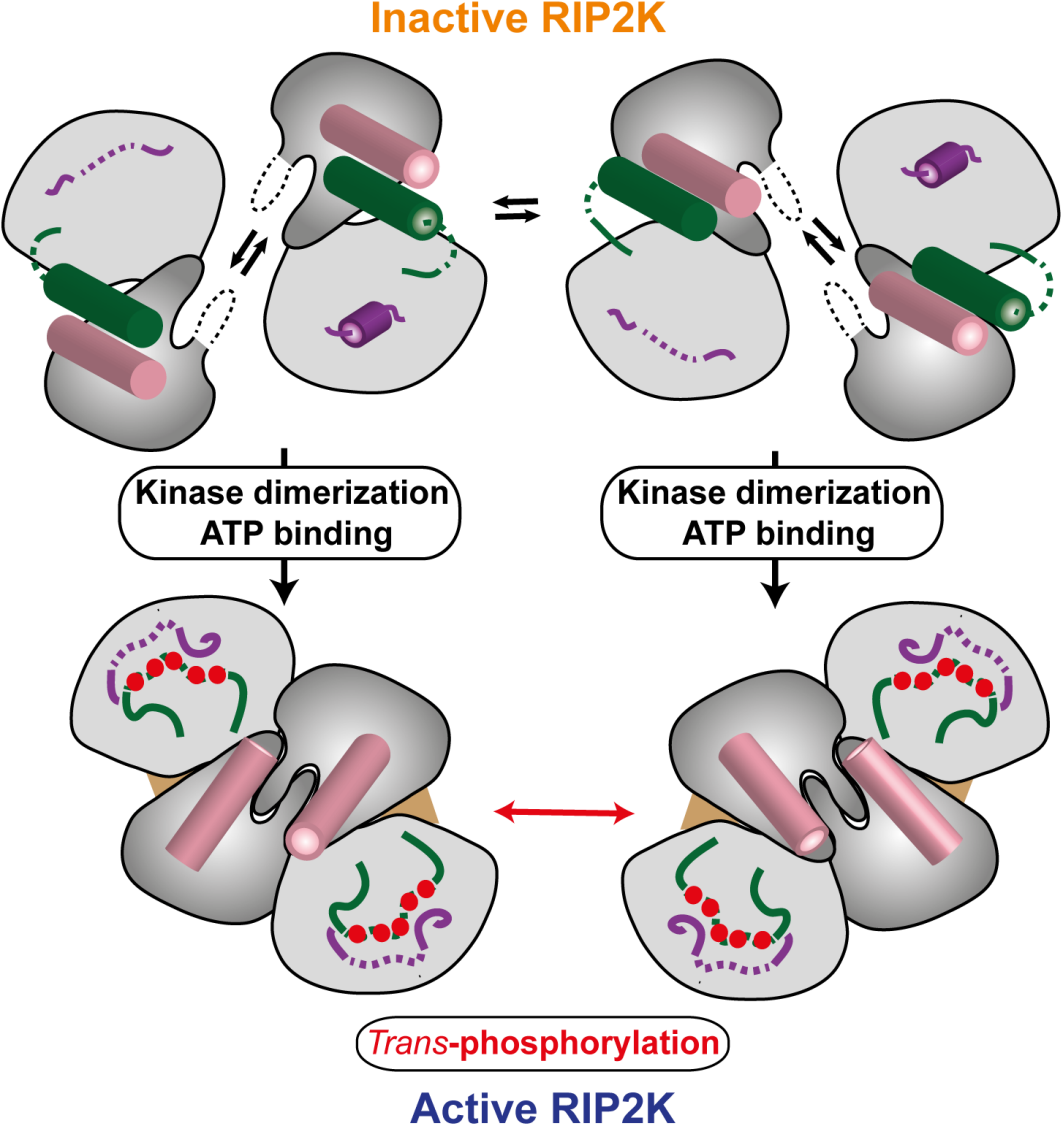
Model of RIP2K activation (see text).

RIP2K activation is stabilized by auto-phosphorylation, which induces a striking change in the AS from a helix to a disordered loop. Our MS results confirm that the RIP2K AS is phosphorylated on Ser174, Ser176 and Ser178 and on Ser180 or Ser181, while Ser168 remains un-phosphorylated. Furthermore, our radioactive kinase assay data show that both inactive RIP2K mutants (RIP2K_K47R_ and RIP2K_D146N_) can be phosphorylated by full length RIP2, indicating that this is *trans*-phosphorylation rather than *cis*-phosphorylation. Comparison of the inactive and active states reveals the importance of coupled kinase dimerization and auto-phosphorylation in promoting enzyme activation. The asymmetric unit of RIP2K_K47R_ suggests a possible model of *trans*-phosphorylation, where the RIP2K phosphorylation occurs between two side-by-side un-phosphorylated RIP2K dimers (Fig 8). The ability of RIP2K_K47R_ to form tetramers at high concentration might suggest that *in vivo* the formation of the prone-to-autophosphorylation complex is caused by increasing the local concentration of kinase domains and consequently forcing dimerization, without previous phosphorylation at the activation loop. In support of this, AUC analyses show that RIP2K_K47R_ has a tendency to form tetramers and RIP2K_D146N_ structure show an active dimer without phosphorylation at AS. Once RIP2K has dimerised, it assumed an active conformation and first phosphorylation event happens *in trans* between kinase dimers (Fig 8). Kinase activation by upstream oligomerisation or crowding events has already been described [68, 76–78]. In the context of the entire RIP2 protein, the oligomerization event might be driven by NOD2 oligomerisation.

Comparison of our RIP2K structures reveals differences in the conformation of the Lys209 loop. In all structures, Lys209, at the C-terminal end of the Lys209 loop (residues 200–210), is well-ordered with its side-chain engaged in hydrogen bonds with the main-chain carbonyl-oxygens of P277 and R280 thus stabilising a helical turn conformation of residues 277–280. Generally the rest of the Lys209 loop is disordered, notably in the active, AMPPCP bound, structures. However in chain A of the RIP2K_K47R_ model the loop is structured and contains a short helix (Fig 3A). A similarly structured loop is observed in one monomer of the RIP2K_D146N_-STAU bound dimer and in recently described structures with bound inhibitors ponatinib, SB2 or biphenyl-sulfonamide (PDB codes: 4C8B, 5AR4, 5AR8 respectively) [38, 39]. These observations suggest that the Lys209 loop is in a dynamic equilibrium between a helical or disordered conformation. During kinase activation, the AS moves from being packed against Helix αC to being close to the Lys209 loop. Intriguingly, the proximity of the phosphorylated AS may affect the conformation of the Lys209 loop in such a way that the accessibility of Lys209 to the ubiquitination machinery may be influenced (Fig 8).

The activation of RAF kinases also requires both dimerization and phosphorylation at the AS [42]. Importantly, the structures of the BRAF monomer and dimer reassemble the RIP2K_K47R_ and the RIP2K-AMPPCP dimer, respectively. In particular, their dimeric interfaces are almost identical (S3 Fig) and their Helix αC are in the similar positions (Figs 2C and 3C). Interestingly, the side-by-side dimer interface mutant BRAF_R509H_, which corresponds to RIP2K_R74H_, results in loss of its catalytic activity in cells and it diminishes dimerization of wild-type BRAF [42]. Therefore, we hypothesize that the mechanism of the RIP2K activation is similar to the BRAF. In this scenario, good candidates for RIP2K drug design should promote the swing-out of the Helix αC and cause the dimer break, as for instance achieved by a sulphonamide inhibitor for BRAF [43].

In conclusion, stable dimerization through a reinforced interaction at the N-termini, the Helix αC displacement and the AS phosphorylation are characteristic features of RIP2K activation. Furthermore, our structural and biochemical characterisation of this process opens new perspectives toward the effective design of drugs against severe inflammatory diseases caused by dysfunctional NOD2-RIP2 signalling.

Inactive RIP2K dimer is in equilibrium with its monomeric form. The activation of RIP2K takes place through the coupling of dimerization and auto-phosphorylation. Dimerization stabilizes the interaction at the N-termini and allows the corrected positioning of the Helix αC (pink). Thus, ATP can be bound and trans-phosphorylation can occur between two dimers. Consequently, the phosphorylated AS (green) becomes unstructured and moves closed to the Lys209 loop, which is also disordered. The N-lobe is coloured in dark grey, C-lobe in light grey. Helices are represented as cylinders. Disordered regions are in dashed lines. Phosphorylation sites are represented as red circles, ATP molecules as a brown triangular. Domain colouring is consistent with Figs 2 and 3.

## Supporting information

S1 Fig Sequence alignment of the kinase domain of PKA with RIP2 and several other human kinases. The secondary structure assignment of PKA is shown on top. Relevant kinase domains and residues are highlighted: Gly-rich loop (residues 50–57 in PKA), the β-strand 3 (which contains the invariant Lysine, Lys72 in PKA), Helix αC (which contains the invariant Glutamate, Gln 91 in PKA), the motif HRD (which contains the aspartate proton acceptor, Asp166 in PKA), the highly conserved triplets DFG and APE which flank the Activation Segment (Asp184-Phe185-Gly186 and Ala 206-Pro-207-Glu208 in PKA).Residues belonging to the Magnesium binding loop are highlighted with a black dashed line. Residues belonging to the R- and C-spines are highlighted in yellow and light blue respectively (Leu106-Leu95-Phe185-Tyr164 in PKA R-spine, Val57-Ala70-Met128-Leu172-Leu173-Ile174-Leu227-Met231 in PKA C-spine) [31–34]. Figure prepared with ESPript 3.0 (http://espript.ibcp.fr/ESPript/cgi-bin/ESPript.cgi) with identical residues highlighted in red, and similar residues written in blue.
(TIF)

S2 Fig Mapping the phosphorylation sites of RIP2K AS using MALDI-TOF MS. A) Sequence of the AS of the RIP2K and its possible phosphorylation sites. B) Phosphopeptides purified by IMAC and analysed by MALDI-TOF MS. We identified a singly phosphorylated peptide at *m/z* 1019.53, and its oxidized form at *m/z* 1035.52, and two doubly phosphorylated peptides at *m/z* 1099.65 (oxidized at 1115.55 and 1131.58) and at 1401.77 (oxidized at 1417.48). Two triply phosphorylated peptides were found at *m/z* 1179.47 (oxidized 1195.62) and 1481.41 (oxidized at 1497.63 and 1513.27). Moreover, a peptide detected at *m/z* 1561.38 was phosphorylated four times (oxidized at 1577.19 and 1593.19). These signals corresponded to two phospho-peptides differently modified. Those at *m/z* 1019.53, 1099.65 and 1179.47 corresponded to the modified 172-MMSLSQSR-179 and three phosphorylation sites were assigned to Ser174, Ser176 and Ser178. The phospho-peptides at *m/z* 1401.77, 1481.41 and 1561.38 corresponded to the phosphorylated 172-MMSLSQSRSSK-182 and the assignment of the three phosphorylation sites (Ser174, Ser176 and Ser178) was further confirmed. A fourth phosphorylation site (present in the phospho-peptide at *m/z* 1561.38) could correspond either to Ser180 or to Ser181. LC-MS/MS analyses on the phosphorylated 172-MMSLSQSR-179 and 172-MMSLSQSRSSK-182 further confirmed the assignment of the Ser174, Ser176 and Ser178 modification sites (data not shown). The presence of additional phosphorylated amino acids within 172-MMS…GQK-203 was suggested by LC-MS/MS experiments, but the exact sites of modification could not be assigned. Overall, the RIP2K AS is phosphorylated on Ser174, Ser176 and Ser178 and on Ser180 or Ser181.
(TIF)

S3 Fig Active and inactive states of BRAF kinase domain. Ribbon diagrams of BRAF kinase domain (A) the active conformation (PDB code:1UWH), (B) the inactive conformation (PDB code: 4WO5) and (C) the side-by-side dimer. N- and C-lobes are represented in dark and light grey respectively. Labelling is consistent with Fig 2. (D) The inset highlights the interactions at the N-termini in the active BRAF dimer. Mutations at Arg509 destabilise the BRAF dimer.
(TIF)

S4 Fig Electron density map of AS in active and inactive conformation. The 2Fo-Fc map is shown as counter level at 1.0 σ. Domain colouring is the same as in Fig 5.
(TIF)

S1 Table Sequence of the recombinant proteins used in this study. Also shown is the associated experimental molecular weight (MW) obtained by analysing intact proteins by LC/ESI-MS. The MWs shown refer to the un-phosphorylated form.
(PDF)

S2 Table Similarity between the RIP2K in the active or inactive state with other kinase structures. The calculations were run with Superpose [69], using the option “*Secondary structure matching*”. PDB codes are shown within parenthesis.
(PDF)
