# Supplementary figures and images for: Structures of the inactive and active states of RIP2 kinase inform on the mechanism of activation
Source: PLoS One. 2017 May 18;12(5):e0177161. doi: 10.1371/journal.pone.0177161 (PMC5436651; doi:10.1371/journal.pone.0177161)

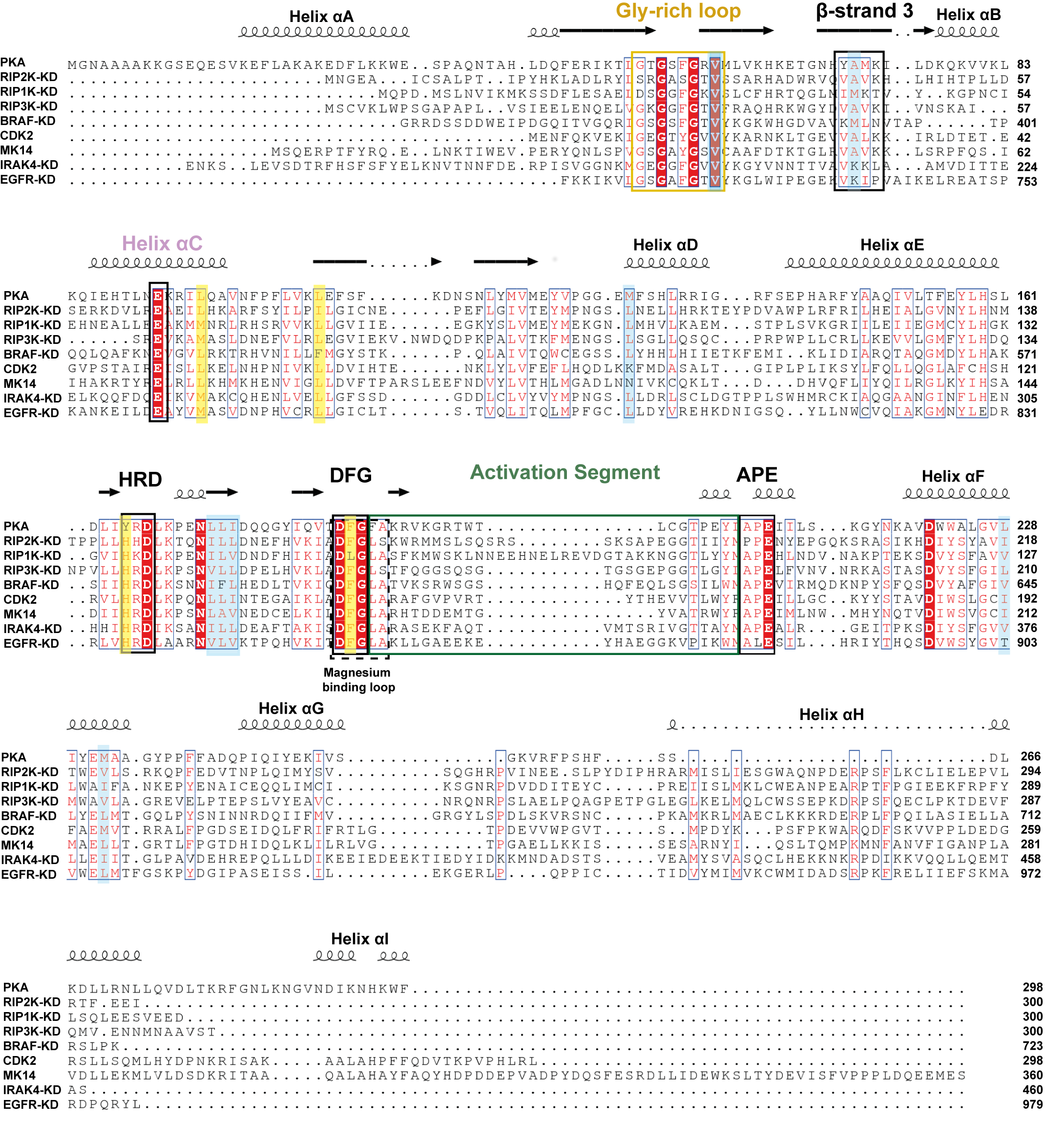

Supplement: S1 Fig — The secondary structure assignment of PKA is shown on top. Relevant kinase domains and residues are highlighted: Gly-rich loop (residues 50–57 in PKA), the β-strand 3 (which contains the invariant Lysine, Lys72 in PKA), Helix αC (which contains the invariant Glutamate, Gln 91 in PKA), the motif HRD (which contains the aspartate proton acceptor, Asp166 in PKA), the highly conserved triplets DFG and APE which flank the Activation Segment (Asp184-Phe185-Gly186 and Ala 206-Pro-207-Glu208 in PKA).Residues belonging to the Magnesium binding loop are highlighted with a black dashed line. Residues belonging to the R- and C-spines are highlighted in yellow and light blue respectively (Leu106-Leu95-Phe185-Tyr164 in PKA R-spine, Val57-Ala70-Met128-Leu172-Leu173-Ile174-Leu227-Met231 in PKA C-spine) [31–34]. Figure prepared with ESPript 3.0 (http://espript.ibcp.fr/ESPript/cgi-bin/ESPript.cgi) with identical residues highlighted in red, and similar residues written in blue. (TIF) [file pone.0177161.s001.tif]

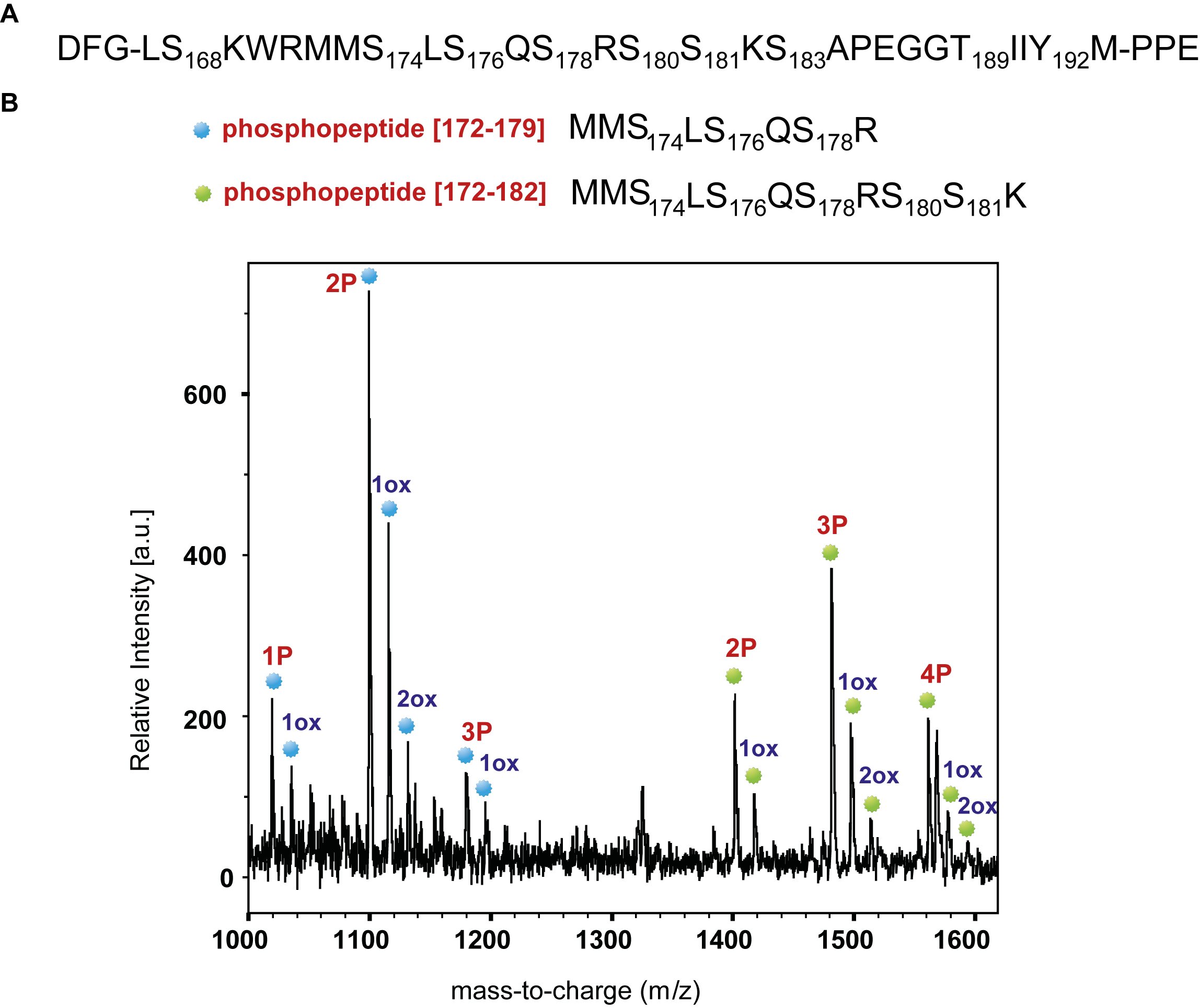

Supplement: S2 Fig — A) Sequence of the AS of the RIP2K and its possible phosphorylation sites. B) Phosphopeptides purified by IMAC and analysed by MALDI-TOF MS. We identified a singly phosphorylated peptide at m/z 1019.53, and its oxidized form at m/z 1035.52, and two doubly phosphorylated peptides at m/z 1099.65 (oxidized at 1115.55 and 1131.58) and at 1401.77 (oxidized at 1417.48). Two triply phosphorylated peptides were found at m/z 1179.47 (oxidized 1195.62) and 1481.41 (oxidized at 1497.63 and 1513.27). Moreover, a peptide detected at m/z 1561.38 was phosphorylated four times (oxidized at 1577.19 and 1593.19). These signals corresponded to two phospho-peptides differently modified. Those at m/z 1019.53, 1099.65 and 1179.47 corresponded to the modified 172-MMSLSQSR-179 and three phosphorylation sites were assigned to Ser174, Ser176 and Ser178. The phospho-peptides at m/z 1401.77, 1481.41 and 1561.38 corresponded to the phosphorylated 172-MMSLSQSRSSK-182 and the assignment of the three phosphorylation sites (Ser174, Ser176 and Ser178) was further confirmed. A fourth phosphorylation site (present in the phospho-peptide at m/z 1561.38) could correspond either to Ser180 or to Ser181. LC-MS/MS analyses on the phosphorylated 172-MMSLSQSR-179 and 172-MMSLSQSRSSK-182 further confirmed the assignment of the Ser174, Ser176 and Ser178 modification sites (data not shown). The presence of additional phosphorylated amino acids within 172-MMS…GQK-203 was suggested by LC-MS/MS experiments, but the exact sites of modification could not be assigned. Overall, the RIP2K AS is phosphorylated on Ser174, Ser176 and Ser178 and on Ser180 or Ser181. (TIF) [file pone.0177161.s002.tif]

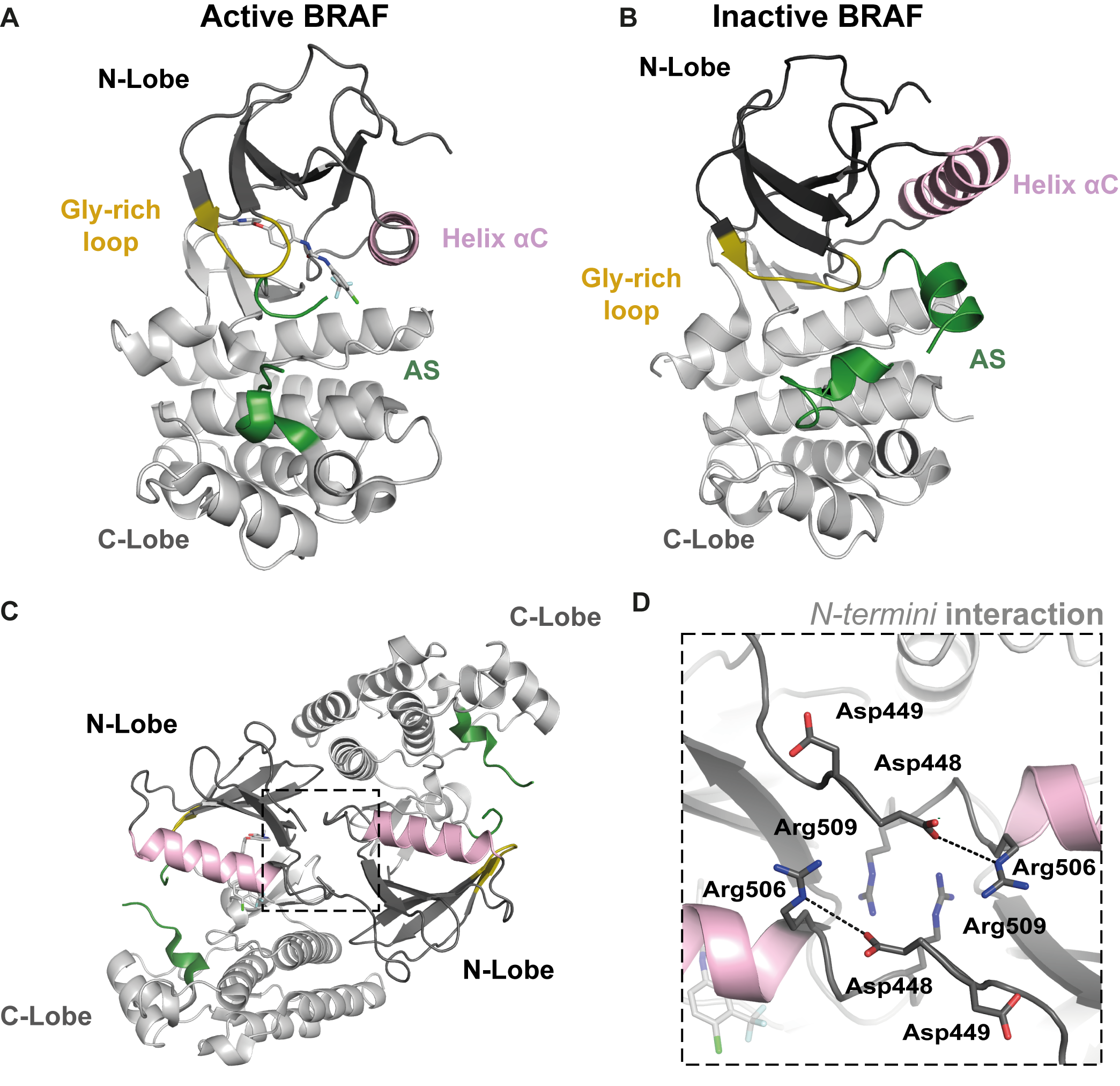

Supplement: S3 Fig — Ribbon diagrams of BRAF kinase domain (A) the active conformation (PDB code:1UWH), (B) the inactive conformation (PDB code: 4WO5) and (C) the side-by-side dimer. N- and C-lobes are represented in dark and light grey respectively. Labelling is consistent with Fig 2. (D) The inset highlights the interactions at the N-termini in the active BRAF dimer. Mutations at Arg509 destabilise the BRAF dimer. (TIF) [file pone.0177161.s003.tif]

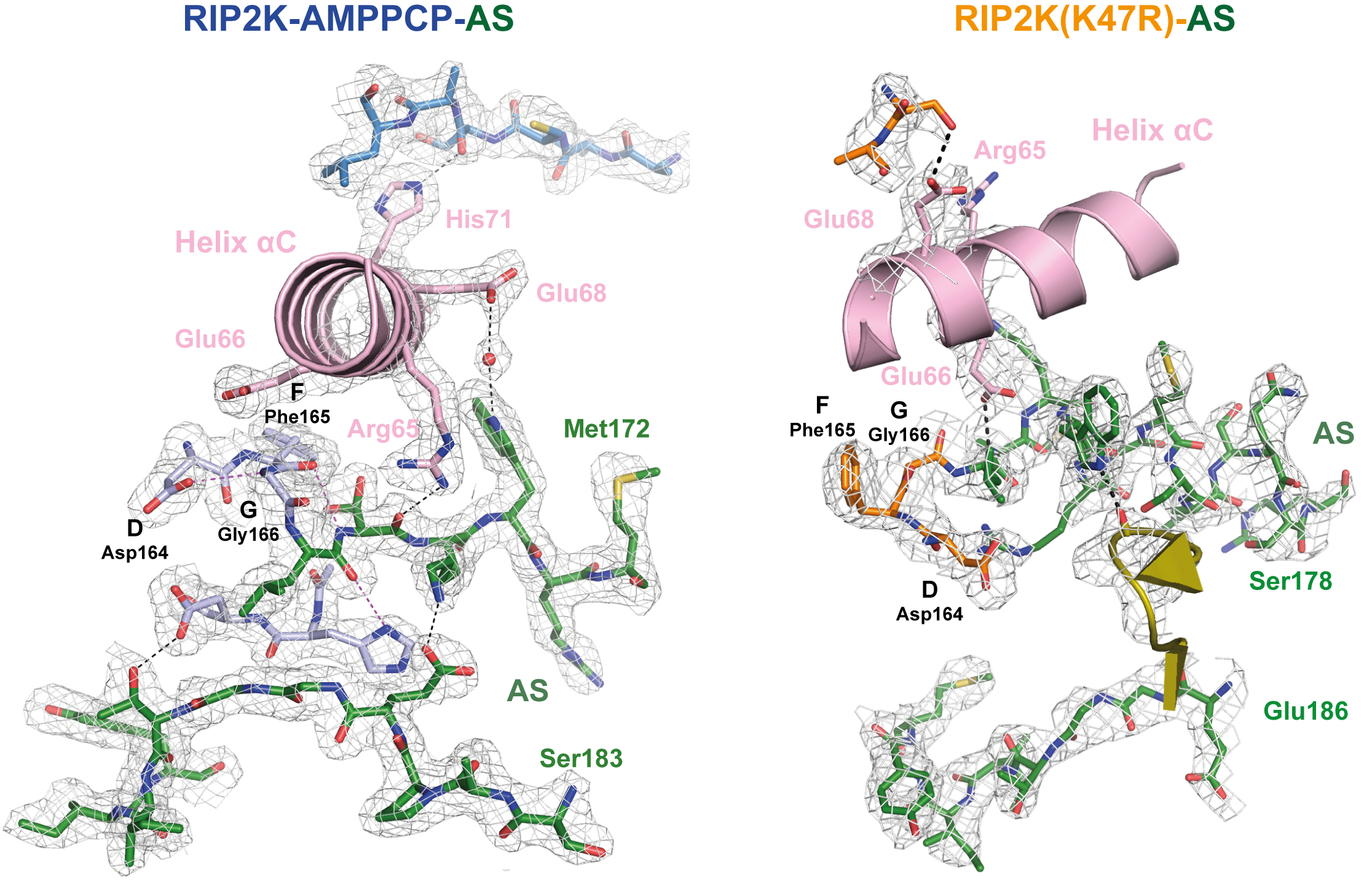

Supplement: S4 Fig — The 2Fo-Fc map is shown as counter level at 1.0 σ. Domain colouring is the same as in Fig 5. (TIF) [file pone.0177161.s004.tif]
