# Supplementary material for: Structures of the inactive and active states of RIP2 kinase inform on the mechanism of activation
Source: PLoS One. 2017 May 18;12(5):e0177161. doi: 10.1371/journal.pone.0177161 (PMC5436651; doi:10.1371/journal.pone.0177161)
Supplement: S1 Table — Also shown is the associated experimental molecular weight (MW) obtained by analysing intact proteins by LC/ESI-MS. The MWs shown refer to the un-phosphorylated form. (PDF) [file pone.0177161.s005.pdf]

Protein name	Protein Sequence	MW (Da)
**RIP2K**	GAMAMNGEAICSALPTIPYHKLADLRYLSRGASGTVSSARHADWRVQVAV KHLHIHTPLLDSERKDVLREAEILHKARFSYILPILGICNEPEFLGIVTEYMPN GSLNELLHRKTEYPDVAWPLRFRILHEIALGVNYLHNMTPPLLHHDLKTQNI LLDNEFHVKIADFGLSKWRMMSLSQSRSSKSAPEGGTIIYMPPENYEPGQKS RASIKHDIYSYAVITWEVLSRKQPFEDVTNPLQIMYSVSQGHRPVINEESLPY DIPHRARMISLIESGWAQNPDERPSFLKCLIELEPVLRTFEEI	34765.01
**RIP2K$_{L294F}$**	GAMAMNGEAICSALPTIPYHKLADLRYLSRGASGTVSSARHADWRVQVAV KHLHIHTPLLDSERKDVLREAEILHKARFSYILPILGICNEPEFLGIVTEYMPN GSLNELLHRKTEYPDVAWPLRFRILHEIALGVNYLHNMTPPLLHHDLKTQNI LLDNEFHVKIADFGLSKWRMMSLSQSRSSKSAPEGGTIIYMPPENYEPGQKS RASIKHDIYSYAVITWEVLSRKQPFEDVTNPLQIMYSVSQGHRPVINEESLPY DIPHRARMISLIESGWAQNPDERPSFLKCLIELEPV**F**RTFEEI	34798.7
**RIP2K$_{K47R}$**	GAMAMNGEAICSALPTIPYHKLADLRYLSRGASGTVSSARHADWRVQVAV **R**HLHIHTPLLDSERKDVLREAEILHKARFSYILPILGICNEPEFLGIVTEYMPN GSLNELLHRKTEYPDVAWPLRFRILHEIALGVNYLHNMTPPLLHHDLKTQNI LLDNEFHVKIADFGLSKWRMMSLSQSRSSKSAPEGGTIIYMPPENYEPGQKS RASIKHDIYSYAVITWEVLSRKQPFEDVTNPLQIMYSVSQGHRPVINEESLPY DIPHRARMISLIESGWAQNPDERPSFLKCLIELEPVLRTFEEI	34793.9
**RIP2K$_{D146N}$**	GAMAMNGEAICSALPTIPYHKLADLRYLSRGASGTVSSARHADWRVQVAV KHLHIHTPLLDSERKDVLREAEILHKARFSYILPILGICNEPEFLGIVTEYMPN GSLNELLHRKTEYPDVAWPLRFRILHEIALGVNYLHNMTPPLLHH**N**LKTQNI LLDNEFHVKIADFGLSKWRMMSLSQSRSSKSAPEGGTIIYMPPENYEPGQKS RASIKHDIYSYAVITWEVLSRKQPFEDVTNPLQIMYSVSQGHRPVINEESLPY DIPHRARMISLIESGWAQNPDERPSFLKCLIELEPVLRTFEEI	34764.9
**RIP2K$_{R74A}$**	GAMAMNGEAICSALPTIPYHKLADLRYLSRGASGTVSSARHADWRVQVAV KHLHIHTPLLDSERKDVLREAEILHKA**A**FSYILPILGICNEPEFLGIVTEYMPN GSLNELLHRKTEYPDVAWPLRFRILHEIALGVNYLHNMTPPLLHHDLKTQNI LLDNEFHVKIADFGLSKWRMMSLSQSRSSKSAPEGGTIIYMPPENYEPGQKS RASIKHDIYSYAVITWEVLSRKQPFEDVTNPLQIMYSVSQGHRPVINEESLPY DIPHRARMISLIESGWAQNPDERPSFLKCLIELEPVLRTFEEI	34679.92
**RIP2K$_{R74D}$**	GAMAMNGEAICSALPTIPYHKLADLRYLSRGASGTVSSARHADWRVQVAV KHLHIHTPLLDSERKDVLREAEILHKA**D**FSYILPILGICNEPEFLGIVTEYMPN GSLNELLHRKTEYPDVAWPLRFRILHEIALGVNYLHNMTPPLLHHDLKTQNI LLDNEFHVKIADFGLSKWRMMSLSQSRSSKSAPEGGTIIYMPPENYEPGQKS RASIKHDIYSYAVITWEVLSRKQPFEDVTNPLQIMYSVSQGHRPVINEESLPY DIPHRARMISLIESGWAQNPDERPSFLKCLIELEPVLRTFEEI	34723.93
**RIP2K$_{R74H}$**	GAMAMNGEAICSALPTIPYHKLADLRYLSRGASGTVSSARHADWRVQVAV KHLHIHTPLLDSERKDVLREAEILHKA**F**FSYILPILGICNEPEFLGIVTEYMPN GSLNELLHRKTEYPDVAWPLRFRILHEIALGVNYLHNMTPPLLHHDLKTQNI LLDNEFHVKIADFGLSKWRMMSLSQSRSSKSAPEGGTIIYMPPENYEPGQKS RASIKHDIYSYAVITWEVLSRKQPFEDVTNPLQIMYSVSQGHRPVINEESLPY DIPHRARMISLIESGWAQNPDERPSFLKCLIELEPVLRTFEEI	34746.28
