# Supplementary material for: Structures of the inactive and active states of RIP2 kinase inform on the mechanism of activation
Source: PLoS One. 2017 May 18;12(5):e0177161. doi: 10.1371/journal.pone.0177161 (PMC5436651; doi:10.1371/journal.pone.0177161)
Supplement: S2 Table — The calculations were run with Superpose [69], using the option “Secondary structure matching”. PDB codes are shown within parenthesis. (PDF) [file pone.0177161.s006.pdf]

	Active RIP2K (RIP2K-AMPPCP)		Inactive RIP2K ($RIP2K_{K47R}$)
Active BRAF_KD (1UWH)	1.48 Å	Inactive BRAF_KD (4WO5)	1.42 Å
Active PKA (1ATP)	1.70 Å	Inactive PKA (4DFY)	2.81 Å
Active CDK2 (1E9H)	2.02 Å	Inactive CDK2 (1B39)	1.89 Å
Active EGFR_KD (2GS6)	1.95 Å	Active EGFR_KD (2GS7)	1.91 Å
